# Action of the Terminal Complement Pathway on Cell Membranes

**DOI:** 10.1007/s00232-025-00343-6

**Published:** 2025-03-23

**Authors:** Bill H. T. Ho, Bradley A. Spicer, Michelle A. Dunstone

**Affiliations:** Monash Biomedicine Discovery Institute, Department of Biochemistry and Molecular Biology, Monash University, Melbourne, VIC Australia

**Keywords:** Complement system, Membrane attack complex, C5a receptor, Regulation, Inflammation, Anaphylatoxin, Terminal complement pathway, Cell membranes

## Abstract

The complement pathway is one of the most ancient elements of the host's innate response and includes a set of protein effectors that rapidly react against pathogens. The late stages of the complement reaction are broadly categorised into two major outcomes. Firstly, C5a receptors, expressed on membranes of host cells, are activated by C5a to generate pro-inflammatory responses. Secondly, target cells are lysed by a hetero-oligomeric pore known as the membrane attack complex (MAC) that punctures the cellular membrane, causing ion and osmotic flux. Generally, several membrane-bound and soluble inhibitors protect the host membrane from complement damage. This includes inhibitors against the MAC, such as clusterin and CD59. This review addresses the most recent molecular and structural insights behind the activation and modulation of the integral membrane proteins, the C5a receptors (C5aR1 and C5aR2), as well as the regulation of MAC assembly. The second aspect of the review focuses on the molecular basis behind inflammatory diseases that are reflective of failure to regulate the terminal complement effectors. Although each arm is unique in its function, both pathways may share similar outcomes in these diseases. As such, the review outlines potential synergy and crosstalk between C5a receptor activation and MAC-mediated cellular responses.

**Graphical Abstract:**

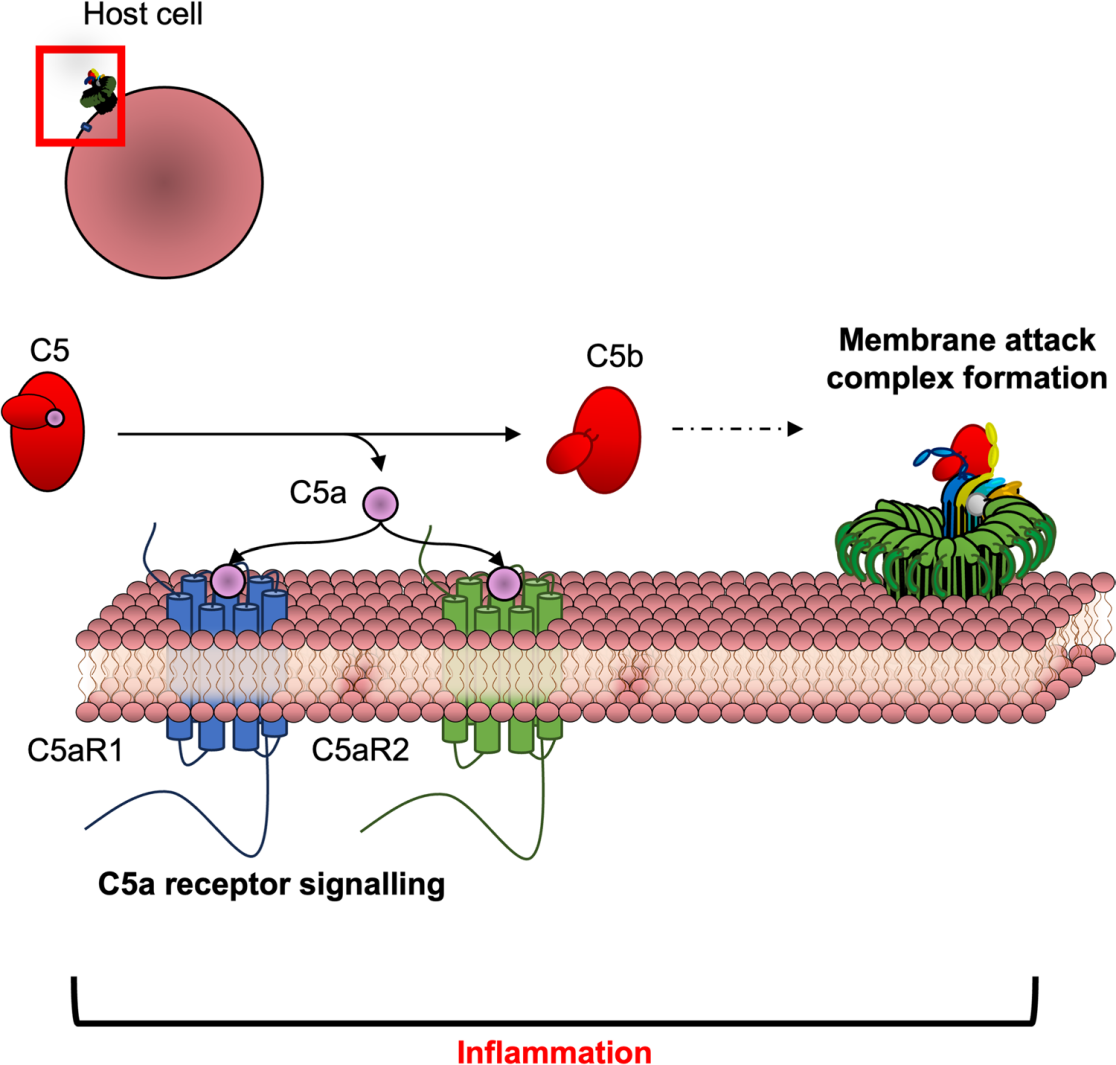

## Introduction

The immune system has remarkable diversity in the strategies for protecting the host organism. Notably, one of these strategies involves triggering an ancient proteolytic pathway known as the complement system on membranes of foreign cells. Complement encompasses a plethora of innate protein effectors and membrane-embedded receptors that facilitate the function of other innate and adaptive arms to boost immunity. Whilst the complement pathway plays a role in immune defence, the pathway is tightly regulated and failure to control its activation results in unwanted consequences such as autoimmunity. Aberrant levels of complement activation on the host result from either a deficiency of regulators or a surplus of activating components and can, therefore, lead to inflammatory diseases (Markiewski and Lambris 2007; Morgan 2016).

**Fig. 1:**
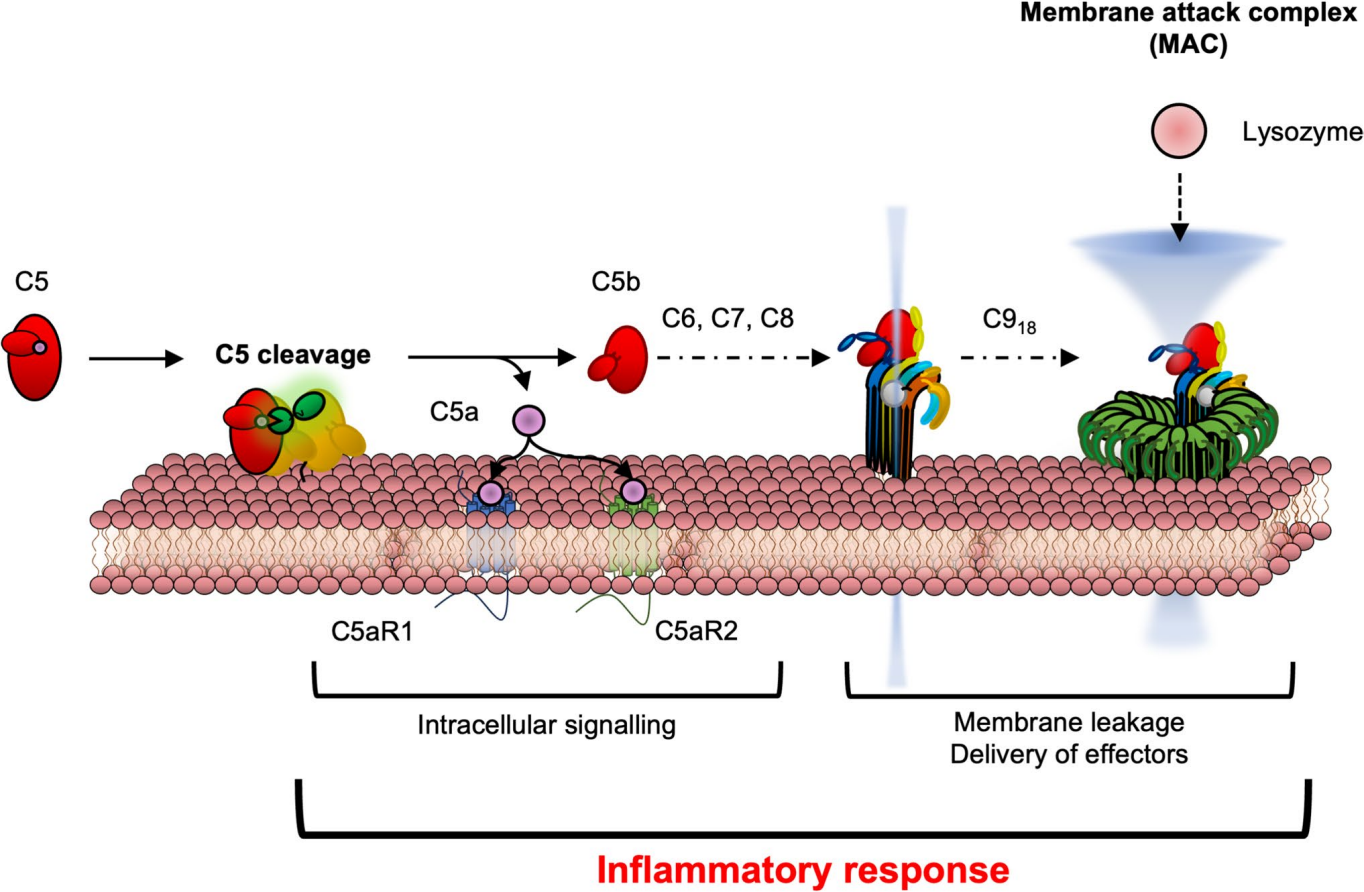
Schematic representation of the terminal complement pathway highlighted in this review

Overall, there are three stages of the complement cascade, each of which has been reviewed extensively concerning their molecular biology. These stages include (1) activation, (2) amplification, and (3) terminal complement pathways (Bayly-Jones et al. 2017; Lachmann 2009; Lee et al. 2008; Noris and Remuzzi 2013). Initially, complement is activated by the classical, lectin or alternative pathways. These pathways facilitate alternative pathway-mediated amplification, ultimately leading to the conversion of C5. Cleavage of C5 marks the terminal stages of complement. The terminal complement pathway, the subject of this review, can promote inflammation by directly interacting with host cell membranes through two distinct effector pathways. Firstly, C5a binds to its receptors to activate highly potent signalling pathways on host cells. Secondly, the membrane attack complex (MAC), a transmembrane hetero-oligomeric pore, spans the membrane on the cells causing osmotic and ion flux, potentially leading to cell lysis (Fig. 1). Collectively, C5a receptor activation and MAC assembly help enhance pro-inflammatory responses in innate cells, leading to the elimination of pathogens but also potentially leading to inflammatory disease. To treat terminal complement-driven inflammatory diseases, recent discoveries and developments of C5a receptor antagonists and MAC-specific inhibitors act on their effectors while retaining the activity of the other.

Here, recent advances in the terminal complement pathway are highlighted by reviewing the molecular and structural insights on the two terminal pathways: (1) the C5a effector pathway and (2) the C5b MAC pathway (Fig. 1). This includes an explanation of how these proteins generate their cellular effects either directly (by permeating membranes) or across the membrane (through signalling). Moreover, this review will explain how the host and recent synthetic inhibitors can modulate these effectors to dull the effects of complement, thereby limiting unintended inflammation.

## Complement: An Ancient Set of Effectors Used to Target Membranes

**Fig. 2:**
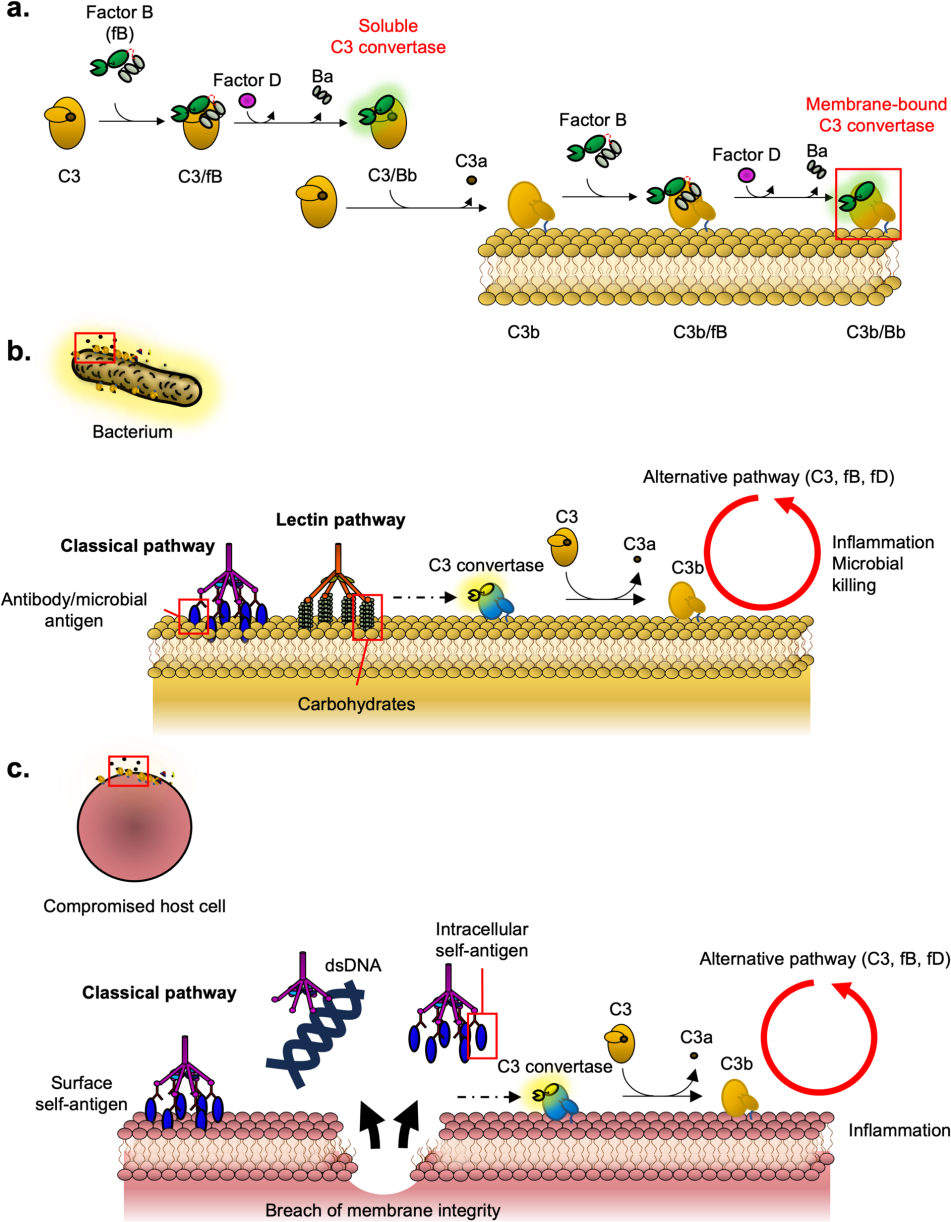
Complement activation directed on surfaces of microbes and host cells. **a** The molecular basis behind alternative pathway activation. Hydrolysed soluble C3 interacts with Factor B and is cleaved by Factor D to form a soluble alternative pathway C3 convertase C3/Bb. The soluble alternative pathway C3 convertase cleaves C3 that binds to membranes, eventually forming the membrane-bound alternative pathway C3 convertase. **b** Complement activation on microbes. The classical and lectin pathway recognises antigens and carbohydrates by antibody and mannose-binding lectin, respectively. This recruits proteases required for forming the classical and lectin pathway C3 convertases (C4b/2a, coloured in blue and yellow). The C3 convertase facilitates C3 cleavage and assists in complement amplification, primarily mediated by the alternative pathway. **c** Complement activation on host cells in the context of autoimmunity. Autoantibodies target self-antigens expressed on the surface and intracellular self-antigens, including double-stranded DNA (dsDNA). This activates complement via the classical pathway, eventually leading to complement amplification via the alternative pathway and inflammation

The complement system is one of the most evolutionarily ancient forms of immunity, providing means to eliminate many threats to the host. The alternative pathway is the oldest known activation arm of complement, found in invertebrates such as sea urchins and horseshoe crabs (Al-Sharif et al. 1998; Tagawa et al. 2012). The alternative pathway is primarily governed by the C3 convertase complex formed by C3b, Factor B and Factor D (Fig. 2A). The soluble C3 convertase forms when Factor B interacts with hydrolysed C3, ultimately forming a proteolytic complex that cleaves C3 into its products C3a and C3b. The formation of C3b exposes an internal thioester that covalently attaches to hydroxyl or sulfuryl groups on membranes and surface proteins on target cells. Attachment of C3b to membranes can be influenced its surface charge, depending on the property of the phospholipid head, thereby demonstrating some degree of specificity to the lipid head groups (Chonn et al. 1991).

Later in evolution, two other protein pathways evolved to facilitate amplification on specific targets. The lectin pathway is the first to have evolved, tracing as early as ascidians and amphioxi (Endo et al. 2003; Sekine et al. 2001). The lectin pathway uses mannose-binding lectins (MBLs) to recognise pathogen-specific carbohydrates present in microbes (pathogen-associated molecular patterns or PAMPs) [extensively reviewed in Endo et al. (2006)]. The second pathway is the classical pathway, which is postulated to have evolved from the lectin pathway (Dodds and Matsushita 2007). The classical pathway uses C1q, which generally recognises antigen-bound antibodies from the adaptive immune system (Fig. 2B). Complexes from these pathways recruit complement scaffold proteins and proteases, which ultimately converge on the formation of lectin/classical pathway C3 convertase (C4b/2a).

Although the immune system is trained to recognise self and non-self, in some circumstances, host cells can present self-surface antigens that generate autoantibodies and cause unnecessary activation of the classical pathway on the host cells. In addition, there are innate factors that can stimulate the PAMP/damage-associated molecular pattern (DAMP)-based pathways on host cells. For example, recognition molecules from the classical and MBL pathways (e.g., serum amyloid P and C-reactive protein) bind to molecules presented by compromised or apoptotic host cells such as double-stranded DNA and exposed oxidised lipids (Fig. 2C) (Bickerstaff et al. 1999; Chang et al. 2002; Flierman and Daha 2007). While this may be beneficial in some cases, such as the clearing of apoptotic cells, it may also give rise to autoimmune diseases, including paroxysmal nocturnal haemoglobinuria (PNH) and systemic lupus erythematosus (section "Significance of the Terminal Complement Pathway in Inflammation").

Following complement activation on either microbes or host cells, C3b is rapidly deposited on the surface of the target cells. This leads to the formation of surface C3 convertases, increasing the level of C3 cleavage and, in effect, promoting a positive feedback cycle. To regulate and remove C3 convertases, the host uses both membrane-bound and soluble regulators that disrupt the positive feedback cycle of C3 convertase formation (section "Complement Membrane Receptors and Regulators Modulate the Complement Cascade on the Surface of Host Cells").

The amplification of complement leads to the addition of C3b to the C3 convertase complex, forming a C5 convertase consisting of either C3b/Bb/C3b (alternative) or C4b/2a/C3b (classical and lectin). The formation of the C5 convertase leads to C5 cleavage. Cleavage of the pivotal protein, C5, forms two proteolytic products, C5a and C5b, diverging into two terminal pathways. C5a binds to its receptors, which are present on the membrane surface of host cells (sections "Molecular Biology of C5a Binding to its Membrane Receptors" and "Structural Insights Behind the Function of C5a Receptors") whereas C5b initiates the formation of the MAC on membranes (section "MAC-Directed Disruption of Target Cells"). Upon pore formation, the MAC can cause direct lysis of pathogenic cells by osmotic flux or delivery of pro-cytolytic effectors such as lysozyme (Heesterbeek et al. 2021).

## Complement Membrane Receptors and Regulators Modulate the Complement Cascade on the Surface of Host Cells

Complement components are recognised and modulated by membrane receptors and regulators expressed on the surface of host cells. Complement receptor (CR) 1 and CR2 are large receptors containing repeat complement control protein (CCP) domains. CR3 and CR4 belong to the integrin superfamily. The complement receptor of the immunoglobulin superfamily (CRIg) contains two domains resembling the immunoglobulin variable and constant domains. CR1-4 and CRIg mainly recognise effectors such as cleavage products of C3, including C3b, inactivated C3b (iC3b) and C3c. Following ligand binding, CR1-4 and CRIg receptors are generally involved in the clearance of complement products, immune regulation, and phagocytosis (Vandendriessche et al. 2021). Complement receptors also include G protein coupled receptors (GPCRs) that recognise complement anaphylatoxins. The C3a receptor (C3aR) recognises C3a, a cleavage product of C3. Protease-activated receptors 1 and 4 (PAR1 and PAR4) have been found to recognise C4a, a cleavage product of C4 (Wang et al. 2017a). PAR1 and PAR4 are characterised by containing a fused ligand that is activated by a protease, such as thrombin (Vu et al. 1991). Currently, the structural basis behind how PAR1 and PAR4 recognise C4a and mount an immune modulatory response is unclear. Finally, terminal complement receptors C5aR1 and C5aR2 recognise C5a (section "Molecular Biology of C5a Binding to its Membrane Receptors").

In addition to the many complement receptors, there also exists several regulators that downregulate complement activation and amplification. These regulators include membrane co-factor protein (MCP) and decay accelerating factor (DAF), both containing repeat CCP domains that work to inhibit the C3 convertase. Lastly, CD59, clusterin and vitronectin are regulators that inhibit MAC formation.

## Molecular Biology of C5a Binding to its Membrane Receptors

### C5a Binds to Two Receptors: C5aR1 and C5aR2

Cleavage of C5 by the C5 convertase generates products C5a and C5b, each initiating its terminal complement pathway. C5a is a pro-inflammatory anaphylatoxin which produces a response such as an increase in vascular permeability, the release of pro-inflammatory cytokines, altered metabolism and chemotaxis. C5a is a ~ 10 to 12 kDa heat-stable glycopeptide of 74 amino acids, where the primary N-linked glycosylation site is on N64 (Li et al. 2022).

Upon cleavage of C5, the C5a component binds to its canonical GPCR, C5aR1 (also known as CD88), as well as a secondary receptor C5aR2 (also named C5L2 or GPR77). Both C5aR1 and C5aR2 belong to the class A (rhodopsin-like) family of GPCRs, which are characterised as containing seven α-helices that traverse through the phospholipid bilayer. C5aR1 and C5aR2 are distant relatives of C3aR. C3aR is ~ 37 and ~ 33% identical to C5aR1 and C5aR2, respectively.

In humans, both receptors are expressed in most cell types but predominantly in myeloid cells originating from bone marrow. Immunohistochemistry analysis has revealed that both receptors have different expression levels depending on the specific cell type; however, in general, C5aR2 has a lower expression level than C5aR1 (Human Protein Atlas, summarised in Table 1) (The Human Protein Atlas; Thul and Lindskog 2018; Uhlen et al. 2010). In an in vitro context, receptor expression and localisation also differ, depending on the cell type and whether receptors are native or transiently expressed (Hsu et al. 2014; Pundir et al. 2015; Scola et al. 2009).

**Table 1:**
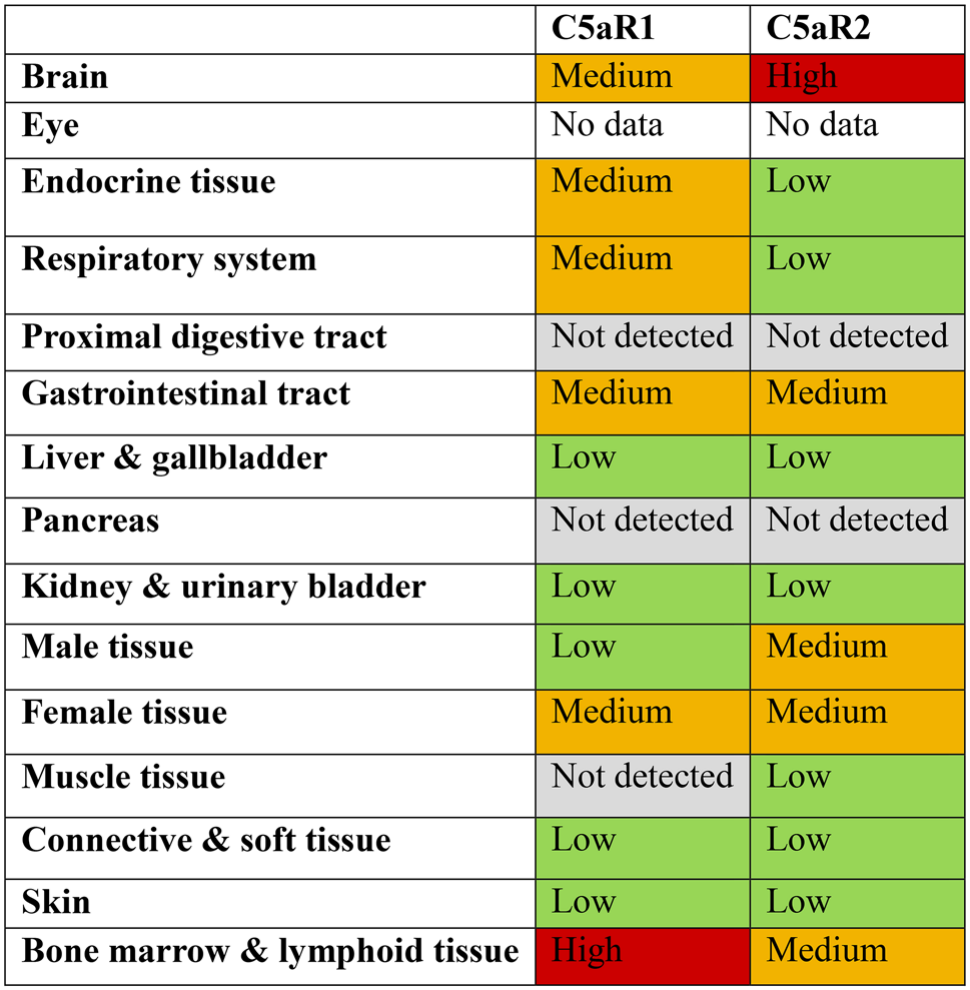
Summary of protein expression of C5aR1 and C5aR2 across different tissues in humans (Thul and Lindskog 2018; Uhlen et al. 2010) (Color figure online)

Note that for the expression level of C5aR2, the Human Protein Atlas (HPA) has annotated this as “uncertain” based on the current strength of evidence provided

**Fig. 3:**
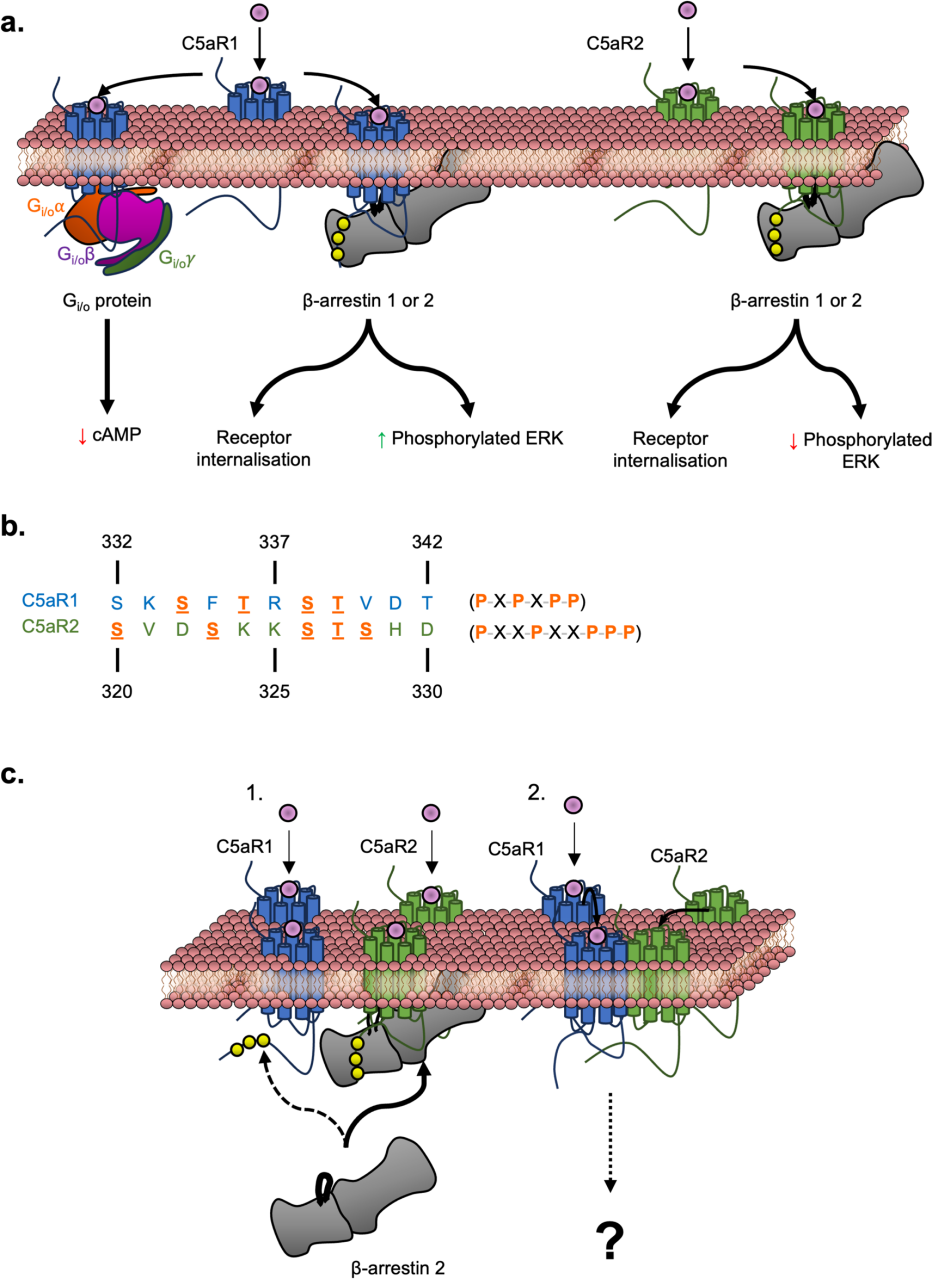
Overview of molecular biology behind C5aR1 and C5aR2 signalling. **a** Stimulation of C5aR1 by C5a results in recruitment of the G_i/o_ heterotrimer (Gαβγ). This G protein family inhibits adenylate cyclase, resulting in decreased cAMP levels. Alternatively, the C-terminal tail of C5aR1 is phosphorylated by a GRK, resulting in the recruitment of β-arrestin. This results in effectors such as C5aR1 internalisation or activation of the MAPK/ERK pathway. C5aR2 exclusively recruits β-arrestins following receptor phosphorylation. Unlike C5aR1, C5aR2 stimulation results in a modest decrease in ERK phosphorylation. **b** Comparison of the putative phosphorylation sites between the C-terminal sequences of C5aR1 and C5aR2. The sites are underlined and in orange, and the respective motifs are outlined on the right of the sequences. **c** Potential pathways in the interplay between C5aR1 and C5aR2. In (1), C5a-stimulated C5aR2 outcompetes C5aR1 for β-arrestin 2 recruitment. In (2), stimulation of one C5aR1 receptor by C5a is sufficient in recruiting a non-stimulated C5aR2 to form the C5aR1/C5aR2 heterodimer. However, the functional significance of this heterodimer is currently unclear

Upon binding and stimulation of C5aR1 by C5a, C5aR1 recruits the heterotrimeric G_i/o_ protein through direct interaction of the Gα subunit with highly conserved amino acid motifs on C5aR1 (N–P–X–X–Y in the seventh transmembrane α-helix and D-R-F/D-R-Y in the third transmembrane α-helix, where X represents any amino acid). This recruitment results in G_i/o_α effectors such as adenylate cyclase inhibition (Taussig et al. 1993). Alternatively, C5aR1 recruits β-arrestin 1 or β-arrestin 2 upon phosphorylation of the intracellular C-terminal tail sequence by an associated G protein receptor kinase (GRK). This β-arrestin recruitment effectively stops G protein-mediated signalling. In addition, an alternative network of arrestin-mediated effectors is initiated, such as clathrin-mediated internalisation or arrestin-mediated signalling pathways such as MAPK/ERK (Fig. 3A) (Jean-Charles et al. 2017; Pandey et al. 2021).

### C5a desArg: A Modified C5a Product of Regulation or Inflammation?

One of the inherent physiological processes to limit C5a activity involves rapid conversion by circulating enzymes such as carboxypeptidase N and B (CPN and CPB). CPN and CPB are proteases that remove the C-terminal arginine residue, generating C5a desArg. Unlike CPN, which requires a co-factor for activity, CPB requires thrombin to be active (Skidgel and Erdos 2007; Song et al. 2011). In the competitive radioactive ligand binding assay with transfected receptors on rat basophilic leukaemia cells, C5a desArg has an approximate 20-fold weaker affinity to C5aR1 compared to C5a. By comparison, however, C5a desArg only binds four-fold weaker to C5aR2 than C5a (Cain and Monk 2002).

This difference in loss of binding raises interesting questions about the role of C5a desArg in the inflammatory response. Both C5a and C5a desArg have short half-lives in vivo (~ 2 min in rabbits) (Webster et al. 1982). Therefore, under homeostatic conditions, CPN and CPB may have redundant roles due to the expected low concentration of C5a from complement regulation. Indeed, unchallenged CPN-deficient mice are comparable to the wild-type. However, both CPN and CPB are important during infection, where they may dampen C5aR1-mediated inflammation by generating low-affinity C5a desArg. CPN-deficient mice are observed to be susceptible to C5a-mediated shock (Mueller-Ortiz et al. 2009). CPB has also been demonstrated to dampen inflammation in the mouse model of autoimmune arthritis (Song et al. 2011).

Despite the weakened affinity of C5a desArg for its receptors, previous studies have challenged its role as a regulator of inflammation. Indeed, studies have shown that cells exposed to C5a desArg potentially exhibit pro-inflammatory phenotypes. Neutrophils exert an inflammatory response at higher concentrations of C5a desArg (Webster et al. 1980). However, it is not clear which receptor, C5aR1 or C5aR2, is responsible for these phenotypes, as stimulation of C5aR2 with C5a desArg also promotes activation of murine neutrophils (Seiler et al. 2022). In basophils, despite having a lower potency than C5a (between 10- to 100-fold), C5a desArg can stimulate comparable amounts of pro-inflammatory mediators such as leukotrienes, histamines and cytokines such as IL-4 and IL-13 (Burgi et al. 1994; Eglite et al. 2000). Overall, the effectors of this ligand have yet to be fully characterised.

### Differences Between C5aR1 and C5aR2 in Signalling

Given that C5a can bind to both C5aR1 and C5aR2, this has led to some probing questions about the function of each receptor. C5aR2 contains ~ 35% sequence identity to C5aR1, and both genes lie on chromosome 12 in humans (Pandey et al. 2020). Despite the high sequence identity between the two receptors, there are distinct differences in their sequences that reflect their respective functions. Compared to C5aR1, C5aR2 lacks the highly conserved motifs indicative of G protein recruitment, suggesting that C5aR2 does not signal through the G protein signalling pathway (Fig. 3A) (Scola et al. 2009). However, mutating the C5aR1 motifs into the equivalent position on C5aR2 did not result in a gain of G protein mediated activity (Scola et al. 2009). This result suggests that other elements are required to enable G protein activity or that the mutation affects the overall structure of C5aR2, thereby impacting G protein recruitment (Scola et al. 2009).

Accordingly, C5aR2 can only recruit β-arrestins upon receptor phosphorylation by a GRK. One of the downstream pathways following β-arrestin recruitment is the activation of the MAPK/ERK signalling pathway. Another difference between C5aR1 and C5aR2 is how they regulate ERK phosphorylation in the MAPK/ERK pathway. In C5aR1 transfected HEK293T cells, increasing the dosage of C5a results in an increase of ERK phosphorylation. Conversely, with C5aR2, increasing doses of C5a results in a modest decrease in ERK phosphorylation (Fig. 3A) (Bamberg et al. 2010; Croker et al. 2016; Pandey et al. 2021).

While the exact molecular differences behind C5aR1- and C5aR2-mediated ERK phosphorylation are unclear, the phosphorylation motif of these receptors may influence β-arrestin recruitment and modulate its downstream effectors. These motifs are situated in the C-terminal tail of the receptors and contain a stretch of amino acids that reveal a distinct pattern of phosphorylated residues. The importance of the phosphorylation pattern in the motifs has been experimentally established for other GPCRs, where different patterns impact different β-arrestin 1 conformations (Guillien et al. 2023). Consequently, these different patterns and β-arrestin conformations affect the behaviour of ERK downstream (Haider et al. 2022). C5aR1 contains the P–X–P–X–P–P motif, where P represents a potentially phosphorylated serine or threonine, and X represents any amino acid. In contrast, C5aR2 contains a P–X–X–P–X–X–P motif (Fig. 3B), which is also present in the bradykinin-2 receptor, a closely related class A GPCR. Interestingly, bradykinin-2 receptor stimulation reveals similar patterns of ERK phosphorylation in comparison to C5aR2 (Baidya et al. 2020; Premont 2020). However, while it was postulated that β-arrestin 1 is responsible for mediating the decrease in ERK phosphorylation for the bradykinin-2 receptor, this is not the case for C5aR2. β-arrestin 1 knockdown does not impact ERK phosphorylation in C5aR2-transfected HEK293T cells, but β-arrestin 2 knockdown does have a slight effect, highlighting potential shared roles between the two β-arrestins (Pandey et al. 2021).

### Potential Interplay Between Pathways Stimulated by C5aR1 and C5aR2

Since most cell types express both C5aR1 and C5aR2, both receptors are potentially involved in interactions upon C5a stimulation. It is important to consider these interactions to dissect different signalling outcomes confounded by the two receptors. The first factor to consider is the potential for competition between the two receptors for the same β-arrestin (either 1 or 2). Competition studies using bioluminescence resonance energy transfer (BRET) experiments have revealed that C5aR2 outcompetes C5aR1 for the recruitment of β-arrestin 2, although this was performed at a single concentration of C5a (Croker et al. 2014). This suggests that C5aR2 is a stronger recruiter for β-arrestin 2 than C5aR1 (Fig. 3C).

A second factor to consider is the potential for dimer states of the C5a receptors. Currently, there is evidence for the formation of C5aR1 homodimers and C5aR1/C5aR2 heterodimers but not for C5aR2 homodimers (Floyd et al. 2003; Rabiet et al. 2008). This is consistent with the wider GPCR family, where some discrete dimer partners have been observed. For example, the class C GPCRs function as obligate dimers (Ellaithy et al. 2020). Concerning the C5a receptors, C5aR1 has been observed to directly interact with C5aR2 based on proximity assays. In this case, a BRET assay reveals that, upon stimulation of C5a, there is an increase in C5aR1/C5aR2 heterodimers (Croker et al. 2013). Interestingly, C5a but not C5a desArg induces C5aR1/C5aR2 heterodimer formation (Croker et al. 2013, 2014). Furthermore, the addition of C5aR1 antagonist PMX53 resulted in a decrease of C5aR1/C5aR2 heterodimers, suggesting that C5aR1 stimulation is sufficient for C5aR1/C5aR2 heterodimerisation (Fig. 3C).

There have been studies that attempted to explain the physiological significance behind C5a receptor heterodimerisation. A study has hypothesised that direct interaction with C5aR2 is required for C5aR1-mediated ERK signalling (Hsu et al. 2014). Interestingly, C5aR2 knockout bone marrow-derived macrophages show a loss in ERK phosphorylation in comparison to the wild-type, suggesting that C5aR2 may play a role in facilitating C5aR1-mediated ERK phosphorylation. However, there are many questions regarding the exact molecular mechanism behind this process. Firstly, the precise order of molecular events taking place to enable ERK phosphorylation is unclear. Additionally, whether C5aR1/C5aR2 heterodimerisation is involved is undetermined. Overall, the physiological relevance of C5aR1/C5aR2 heterodimerisation remains largely unknown.

### Elusive Role of C5aR2

In general, there is limited knowledge behind both the intracellular signalling of C5aR2 and the cellular phenotypic outcomes of C5aR2 signalling. Shortly after discovering the protein, the first hypothesis was that C5aR2 acts as a decoy receptor. This concept is similar to the atypical chemokine receptors (ACKRs 1–4). These chemokine receptors contribute to scavenging excess chemokines, thereby inhibiting cell migration and dampening the chemokine-induced cell response (Bonecchi and Graham 2016; Comerford and McColl 2024). Supporting this hypothesis, C5aR2 is also responsible for trafficking receptor-bound C5a for proteasomal degradation and decreases the local concentration of C5a for C5aR1 activation on the membrane surface (Scola et al. 2009). C5aR2 is also later found to regulate C5aR1 by other means. This includes the regulation of C5aR1-mediated infiltration to the site of intestinal reperfusion injury in mice (Wu et al. 2020). Furthermore, stimulating human monocyte-derived macrophages (HMDMs) with C5aR2 agonists also inhibit C5aR1 effectors, resulting in a decrease of pro-inflammatory cytokines including IL-6 and TNF-α (Li et al. 2020).

However, recent emerging reports challenge the decoy receptor theory, showing that C5aR2 has the potential to induce pro-inflammatory responses that are distinct from C5aR1 activation (Miyabe et al. 2019; Pundir et al. 2015; Seiler et al. 2023; Vijayan et al. 2014; Zhang et al. 2020a). The inflammatory effect of C5aR2 stimulation is apparent when different disease models are tested (murine model of epidermolysis bullosa acquisita versus intestinal reperfusion injury) (Seiler et al. 2023), species of macrophages are studied (murine instead of human), and effects on other cell types are explored. Overall, these results contrast with the regulatory role of C5aR2.

## Structural Insights Behind the Function of C5a Receptors

The recent plethora of C5aR1 structures has shed insights into C5aR1 function, which may help elucidate the function of its close homologue, C5aR2. In essence, there are two major structural elements required to explain the C5a receptor function; one is how C5a binds to the receptor, and the second is how the receptor can interface with intracellular effectors (i.e., G protein or β-arrestin) of cell signalling in the cytosol. A third aspect to discuss is how synthetic agonists and antagonists interact with C5aR1 to modulate signalling.

While the structure of C5a has been known for some time, it is the structure of the membrane-embedded C5a receptors that are of most interest (Zhang et al. 1997). Recently, structures of the various states of C5aR1 have been elucidated (Table 2). These models include unbound C5a and, more recently, C5aR1 bound to antagonists, as well as C5a and other non-native ligands in complex with C5aR1 and G_i/o_ protein. Additionally, structures of β-arrestin bound to a phosphopeptide of C5aR1 provides insights into how the C-terminal tail of C5aR1 locks into the intracellular effector.

**Table 2:** List of C5aR1 structures

Structure	Ligands	Other key components	Accession Code	Resolution	Method	References
Inactive C5aR1	NDT9513727 (allosteric antagonist)	N/A	PDB: 5O9H	2.70 Å	X-Ray crystallography	Robertson et al. (2018)
	PMX53 (orthosteric antagonist) & NDT9513727 (allosteric antagonist)	N/A	PDB: 6C1Q	2.90 Å	X-Ray crystallography	Liu et al. (2018)
	PMX53 (orthosteric antagonist) & Avacopan (allosteric antagonist)	N/A	PDB: 6C1R	2.20 Å	X-Ray crystallography	
C5aR1 & G_i/o_ complex	C5a/C5a^peptide^/BM213 (full/partial agonists)	G_i1_	PDB: 7Y64/7Y65/7Y66 EMD: 33633/32634/33635	3.20 Å/2.90 Å/2.80 Å	Cryo-EM	Feng et al. (2023)
	C089 (orthosteric antagonist)	C5aR1(I116A) mutant G_i1_	PDB: 7Y67 EMD: 33636	2.80 Å	Cryo-EM	
	C5a/C5a desArg (full/partial agonists)	Mini G_o_	PDB: 8IA2/8JZZ EMD: 35292/36755	3.21 Å/3.31 Å	Cryo-EM	Yadav et al. (2023)
	C5a/C5a^peptide^ (full/partial agonist)	Murine C5aR1 Mini G_o_	PDB: 8HQC/8HPT EMD: 34947/34943	3.89 Å/3.39 Å	Cryo-EM	
	C5a (full agonist)	G_i_	PDB: 8HK5 EMD: 34846	3.00 Å	Cryo-EM	Wang et al. (2023)
C5aR1 phospho-peptide & β-arrestin complex	N/A	C5aR1 phosphopeptide β-arrestin 1	PDB: 8GO8/8I0N EMD: 34173/35104	3.41 Å/3.26 Å	Cryo-EM	Maharana et al. (2023)
	N/A	C5aR1 phosphopeptide β-arrestin 2	PDB: 8GOO/8I0Z EMD: 34178/35114	4.40 Å/4.33 Å	Cryo-EM	Maharana et al. (2023)

### How Does C5a and C5a desArg Bind to its Receptors?

As previously mentioned, C5aR1 is a class A GPCR, containing seven transmembrane α-helices traversing through the phospholipid bilayer. On the interface facing the extracellular environment is the binding site for C5a. The other major interface lies in the cytosol, where the G_i/o_α subunit docks and recruits Gβγ, resulting in intracellular signalling, such as inhibition of adenylate cyclase.

**Fig. 4:**
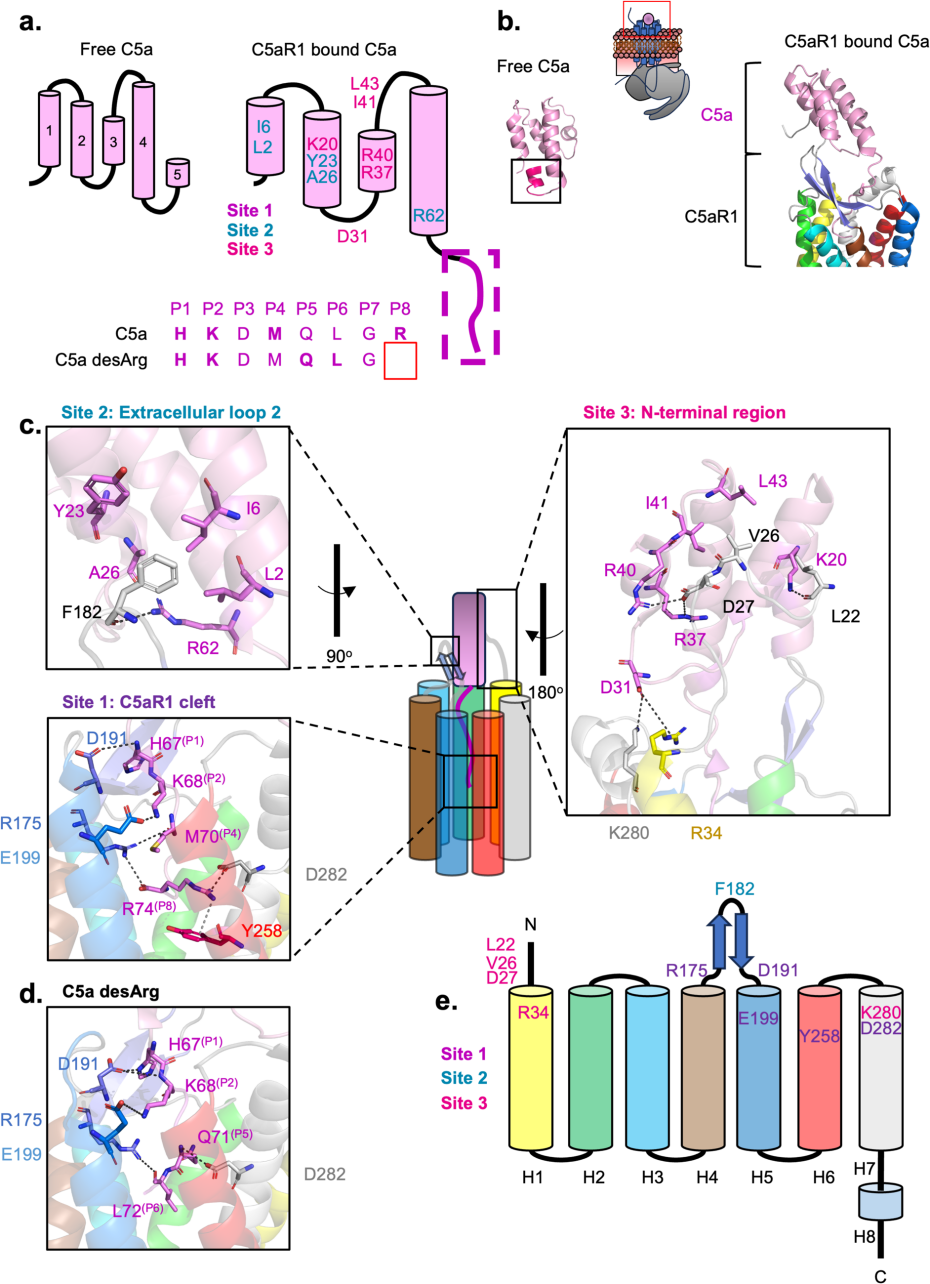
Recognition of C5a and C5a desArg to C5aR1. **a** Simplified schematic of the C5a structure, comparing free C5a and receptor-bound C5a. α-helices one to five are indicated in free C5a, and key interacting residues and their respective sites are indicated in receptor-bound C5a. Below is the sequence of the last 8 residues at the C-terminal end of C5a and the sequence of C5a desArg for comparison. In bold are the residues interacting with C5aR1. **b** The overall structure of free unbound C5a (PDB: 1KJS) compared to C5a bound to C5aR1 (PDB: 7Y64). The last eight residues of C5a that dock into site 1 of C5aR1 are boxed and coloured in magenta in the free C5a structure. **c** Key interacting residues between C5a (in pink) and residues on C5aR1, coloured in their according transmembrane α-helix, in the primary site (site 1) and two facilitative sites (site 2 and site 3). **d** Site 1 interactions between C5a desArg and C5aR1, based on the structure PDB: 8JZZ. **e** Key interacting residues in C5aR1, coloured in their respective sites

Free unbound C5a is a helical bundle containing five α-helices (Fig. 4A, B) (Zhang et al. 1997). Upon binding of C5a to C5aR1, the C-terminal α-helix (H5) unravels to form an extended loop that engages with C5aR1. This extended H5 loop makes extensive interactions with the main groove (site 1) of C5aR1. The second and third sites of C5aR1 lie in the periphery of the receptor, where it helps to wedge C5a into the binding pocket, both increasing its binding affinity and contributing to signalling (Feng et al. 2023; Wang et al. 2023). The second site of C5aR1 involves the extracellular loop 2 (ECL2) (Klco et al. 2005). This ECL2 is also present in chemokine receptors (CCR1, CCR5) and accordingly plays a role in chemokine binding (Shao et al. 2022; Zheng et al. 2017). Thus, C5aR1 shares some topological resemblance to chemokine receptors. The third site is facilitated by the N-terminal strand of C5aR1, interacting with the second and third α-helices of C5a (Fig. 4C).

Within site 1 of C5aR1, electrostatic interactions form between the C-terminal end of C5a and C5aR1, as illustrated in Fig. 4C. Of these interactions, the most relevant involves the stabilisation of the C-terminal arginine (R74) at position 8 (P8) (Fig. 4C). Importantly, cleavage of the C-terminal arginine making the C5a desArg results in reduced interactions, which explains the reduction in binding. In C5a desArg, this stabilisation is lost, changing the conformation of the C5a C-terminal tail (Fig. 4D) (Yadav et al. 2023). Together, the combined structures provide an excellent explanation of the decreased affinity of C5a desArg compared to the potent C5a.

**Fig. 5:**
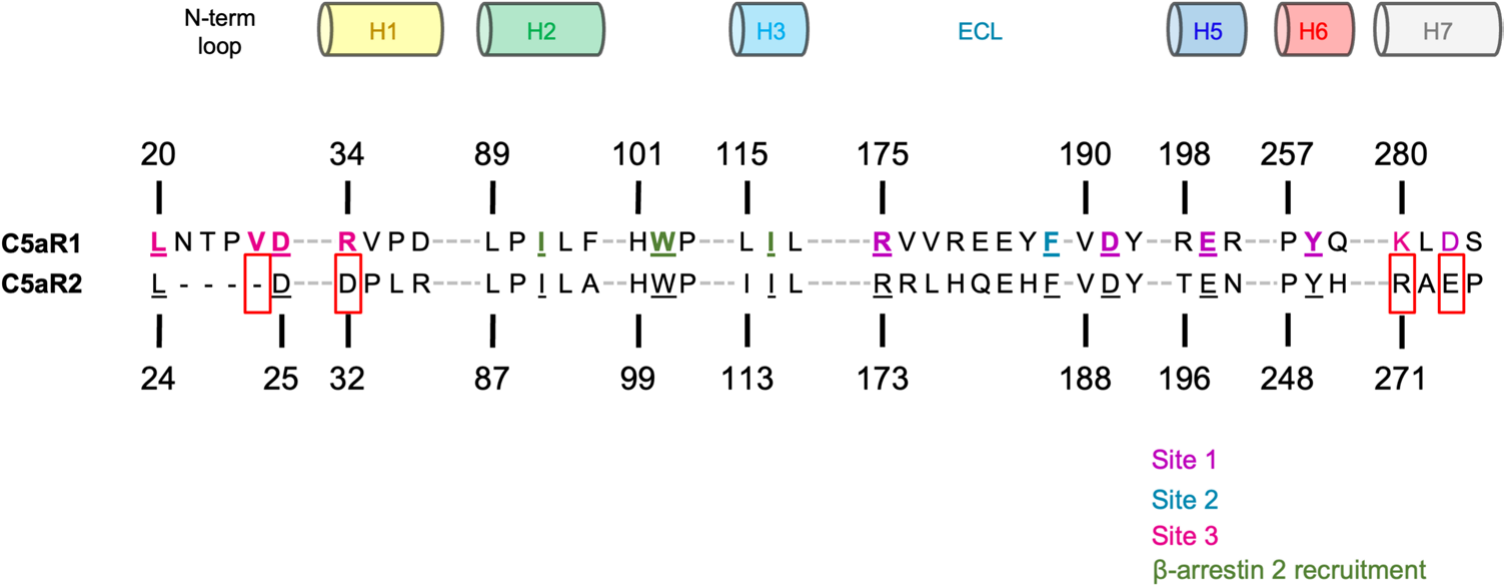
Sequence alignment of C5aR1 and C5aR2, highlighting the key interacting residues based on the structure of C5aR1 bound to C5a. The residues are coloured according to their respective sites. Residues contributing to β-arrestin recruitment are coloured in green. Differences between C5aR1 and C5aR2 are boxed in red

Interestingly, compared to a dramatic decrease in the affinity of C5a desArg for C5aR1 (~ 20-fold), there is only a modest drop in affinity for C5aR2 (~ four-fold) (Scola et al. 2007). In the primary structure, the key residues of C5aR1 that interact with C5a mostly remain conserved for C5aR2 (Fig. 5). Notably, the critical residue D282 is replaced with glutamate in the canonical binding site responsible for binding to the C-terminal arginine of C5a. At the time of writing this review, no studies have performed mutations on this position in C5aR1 to observe whether the glutamate would enhance C5a binding to C5aR1.

Another major difference lies within the third site of C5aR1/R2, where C5a binding is primarily mediated by the N-terminus of the receptors (Figs. 4C, 5). Thus, it is postulated that mutating these residues to the C5aR2 equivalent residues would impact C5a binding. However, a chimeric C5aR2 mutant that has its N-terminus (residues 1–32) replaced with C5aR1 (residues 1–37) did not perturb binding to C5a desArg, disproving that this region solely mediates this specificity (Scola et al. 2007). Therefore, this observation alone does not fully explain why C5a desArg has a similar affinity for C5aR2 compared to full-length C5a.

### Structural Mechanism Behind G_i/o_ Recruitment to C5aR1

**Fig. 6:**
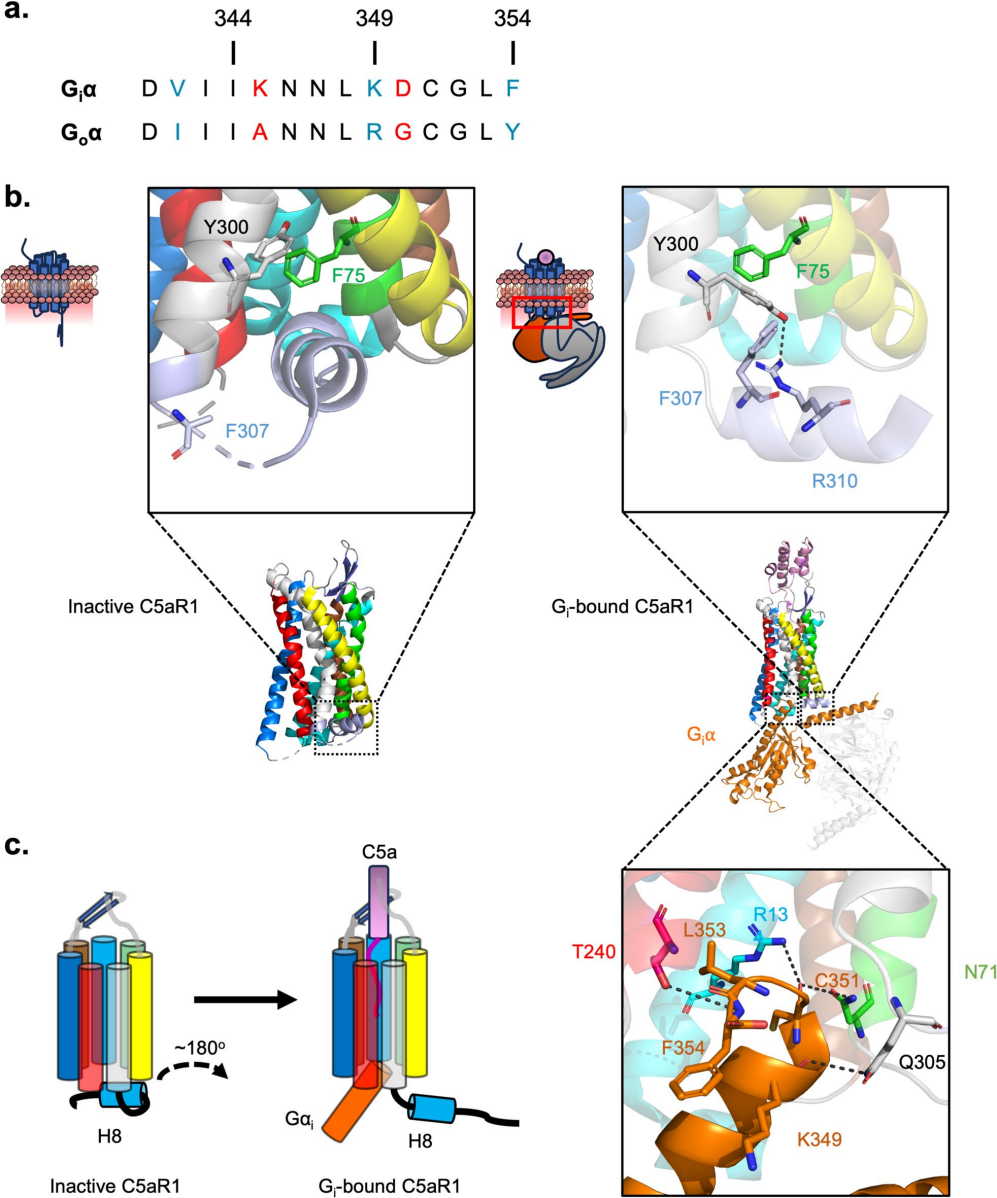
G_i_ recruitment to C5aR1.** a** Sequence alignment of the C-terminal α-helix between the G_i_α and G_o_α subunits. **b** A structural comparison of the eighth intracellular α-helix between inactive C5aR1 (PDB: 6C1Q) and G_i_-bound C5aR1 (PDB: 7Y64). Inside the boxes on top of the structures are regions where the conformational change of the α-helix occurs and the different interactions taking place within C5aR1. Boxed below the structure of G_i_-bound C5aR1 is the specific region where G_i_α interacts with C5aR1, highlighting the key interacting residues between G_i_α (orange) and C5aR1 (coloured in their respective α-helix). **c** Simplified schematic showing the movement of the eighth α-helix following C5a binding

Following receptor stimulation by C5a, the intracellular heterotrimeric G protein is recruited to the receptor. Specifically, the Gα subunit interacts with a cleft of C5aR1 located at the intracellular face of the receptor. For structural determination, different subtypes of the Gα subunit were used, including a truncated version of G_o_ called mini-G_o1_ (Yadav et al. 2023) and a subtype of G_i_, called G_i1_ (Feng et al. 2023; Wang et al. 2023). G_i1_ and G_o1_ are ~ 70% identical, and both members inhibit adenylate cyclase (Jiang and Bajpayee 2009). A comparison of the most recent structures (PDB: 7Y64 and 8IA2) shows that common interactions occur at the C-terminal α-helix of Gα_i1/o1_. Importantly, the sequence of this C-terminal helix is highly conserved between G_i1_ and G_o1_ (Fig. 6A). Here, this α-helix is inserted into the intracellular cleft of C5aR1. Upon interaction of the receptor with C5a, the most significant change in C5aR1 involves the C-terminal intracellular helix 8 (H8), which moves to expose the intracellular cleft of C5aR1 for Gα_i/o_ binding (Fig. 6B, C). The significance of this interaction is highlighted by C5a antagonists, which, upon binding to C5aR1, cause an obstruction of the H8 cleft for Gα_i/o_, providing first-hand structural insights behind G protein antagonism (Fig. 6B).

### Structural Mechanism Behind β-Arrestin 1 and 2 Recruitment to C5aR1

**Fig. 7:**
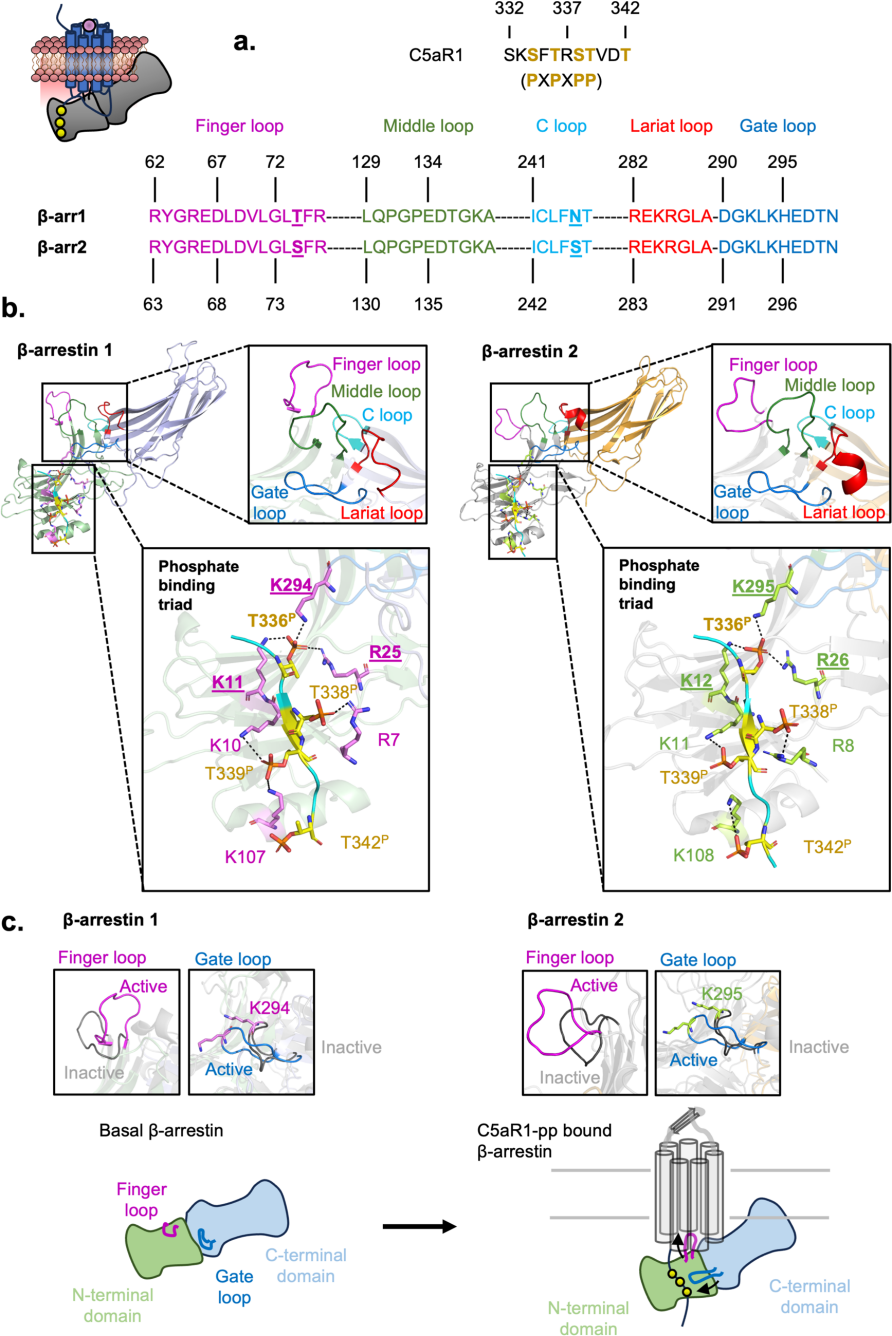
Structural basis behind C5aR1 phosphopeptide binding to β-arrestins 1 and 2. **a** Sequence of the C-terminal tail of C5aR1 with the putative phosphorylated residues coloured in yellow. Below is the sequence alignment of the key receptor interacting loops between β-arrestin 1 and 2. Differences in the sequences are underlined. **b** Structural comparison between C5aR1 phosphopeptide binding to β-arrestin 1 and 2. The loops in the phosphopeptide-bound conformation are indicated in their respective colours (finger loop = magenta, middle loop = green, C loop = cyan, gate loop = blue, lariat loop = red). Below is the N-terminal domain, revealing the lysines (pink for β-arrestin 1 and light green for β-arrestin 2) interacting with the C5aR1 phosphopeptide. **c** Comparison of the finger and gate loops between the resting (PDB: 1G4M and 3P2D) and phosphopeptide-bound β-arrestins (PDB: 8GO8 and 8GOO). Below is a simplified schematic between basal β-arrestin and C5aR1-bound active β-arrestin, including the conformational changes of the finger and gate loops

An intriguing property of C5aR1 is that it can bind β-arrestins as an alternative to binding G proteins. This also has implications for understanding the function of C5aR2, which can only bind β-arrestin. In a process to uncouple G proteins from C5aR1, the C-terminal tail of the receptor is phosphorylated by a GRK that enables β-arrestin binding. As previously mentioned, the C-terminal tail of C5aR1 has a P–X–P–X–P–P phosphorylation motif, which is also found in around a third of the characterised non-olfactory and non-orphan class A GPCRs (Fig. 7A) (Maharana et al. 2023). Phosphorylation of this motif is mediated predominantly by GRK5 and GRK6. In support of this, a combined knockout of the genes encoding GRK5 and GRK6 impacts both β-arrestin 1 and β-arrestin 2 recruitment to C5aR1 overexpressed in HEK293 cells (Pandey et al. 2021).

When looking at the structure, both β-arrestins 1 and 2 contain an N-terminal and a C-terminal bilobed β-sandwich domain. To engage with the receptor, the finger and middle loops of the N-terminal domain, as well as the C and lariat loops of the C-terminal domain, interact with the cleft of the intracellular face of C5aR1 (Huang et al. 2020). Both β-arrestin 1 and 2 contain almost identical sequences of these loops, suggesting their conserved docking mechanism with GPCRs (Fig. 7A, B).

The mechanism of how β-arrestins recognise the phosphorylated motif of C5aR1 and change from a basal to active conformation is a key question in understanding how C5aR1 mediates intracellular signalling. The most recent information comes from a study of an active β-arrestin conformation that is bound to a C5aR1 phosphopeptide (Fig. 7A). Overall, both β-arrestins reveal remarkable similarity in phosphopeptide engagement and conformational changes of both the C-terminal domain and the canonical loops. Within the N-terminal domain, the finger loop is more exposed to allow docking into C5aR1 in comparison to the resting bovine β-arrestin (Han et al. 2001; Zhan et al. 2011). The gate loop contains key residues that are important for stabilising the basal conformation of β-arrestin (Kim et al. 2024). In comparison to the basal conformation, the gate loop in the active conformation subtly shifts to interact with the C5aR1 phosphopeptide (Fig. 7B, C). Thus, movement of the gate loop may contribute to the transition between the basal and active β-arrestin conformations.

Despite the similarities in β-arrestin binding to the phosphopeptide and high sequence identity in the docking residues between β-arrestin 1 and 2, the overall mechanism behind how C5aR1 differentiates between the two β-arrestins is unclear. Two key factors may modulate this process. One factor includes the intracellular concentration and localisation of each β-arrestin. Another factor involves other combinations of phosphorylation patterns in the motif. Different phosphorylation sequences influence β-arrestin binding, which has been the case for other GPCRs such as the rhodopsin receptor, vasopressin 2 receptor, ghrelin receptor type 1a, and β2-adrenergic receptor (Guillien et al. 2023; Mayer et al. 2019). Reflecting this, a key limitation behind the structural study is that the β-arrestins were only coupled to one specific C5aR1 phosphopeptide sequence.

### Concept of Biased Agonism in C5aR1 Signalling

As C5aR1 activation leads to a wide variety of signalling outcomes, several factors lead to activation of different signalling pathways. These include the species studied, availability of ligands, receptor expression levels, different isoforms of G protein, as well as localisation of G protein and β-arrestin 1/2 in host cells. In an attempt to deconvolute the complex repertoire of signalling outcomes, peptide-based agonists of C5aR1 were constructed that function to preferentially activate one intracellular signalling pathway over the other. In this case, when compared to native C5a, these agonists would prefer either C5aR1-mediated G protein recruitment over β-arrestin recruitment or vice versa.

**Fig. 8:**
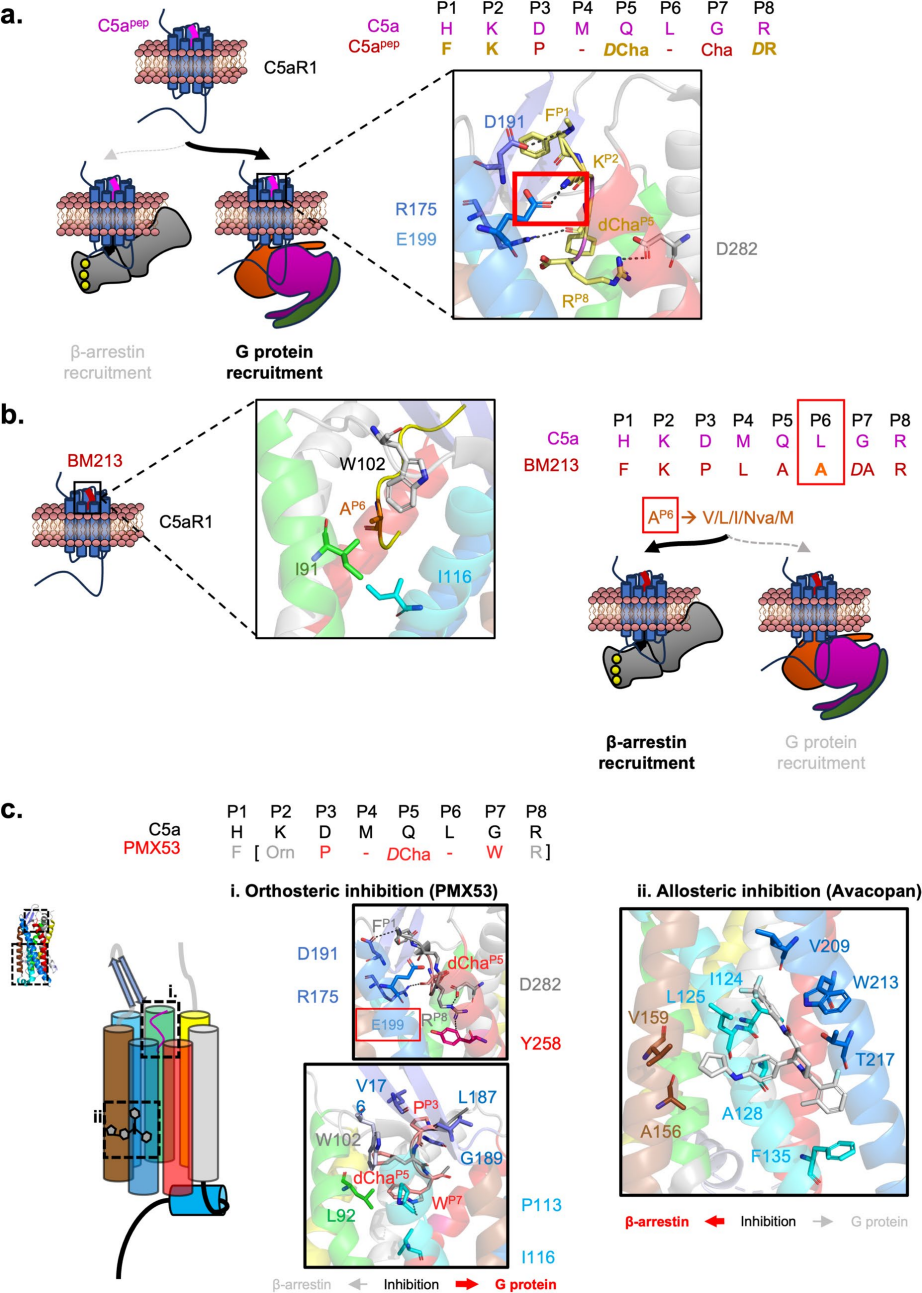
Structural basis behind biased-agonism in C5aR1 activation and inhibition. **a** Key interacting residues between C5a^pep^ (yellow) and C5aR1 resulted in a bias towards G_i_-mediated activity (PDB: 7Y65). **b** Interacting residues between the alanine at P6 on BM213 and hydrophobic residues on C5aR1, coloured in their respective α-helix (PDB: 7Y66). Changing this residue to a more hydrophobic residue, except phenylalanine, results in increased β-arrestin 2 bias. **c** The structural mechanism leading to (i) orthosteric inhibition, mediated by PMX53, and (ii) allosteric inhibition, mediated by Avacopan (PDB: 6C1R). A comparison between the sequences of C5a and PMX53 is shown. This includes the absence in the interaction of E199 in C5aR1 and the hydrophobic interactions between proline, d-Alanine (dCha) and tryptophan at P3, P5 and P7 of PMX53 and hydrophobic residues of C5aR1. As a result, there is a bias in inhibiting G protein recruitment. By contrast, Avacopan interacts with hydrophobic residues on transmembrane helices 3, 4 and 5 of C5aR1, resulting in biased inhibition of β-arrestin recruitment

For example, a C5a peptide (C5a^pep^ or C028), derived from the native sequence of the C-terminal region of C5a, exhibits biased activation of G_i/o_ relative to full-length C5a due to lower levels of β-arrestin 2 recruitment (Feng et al. 2023; Yadav et al. 2023). Based on this observation, it is implied that important interactions occur involving D191 and E199 of C5aR1 to mediate G protein activity. Reflecting this, alanine mutations on these residues had the most impact on G protein activity (Fig. 8A). Recently, C5a^pep^ has also been found to activate C5aR2 and recruit β-arrestin 2 (Li et al. 2021). Therefore, further modification of C5a^pep^ may be beneficial as a tool to further elucidate C5aR2-specific β-arrestin bias.

Biased agonism can also be explained by comparing the activity of C5a^pep^ between different species of C5aR1. In comparison to human C5aR1, there is a significant reduction of β-arrestin 1 and 2 recruitment in murine C5aR1 when stimulated with C5a^pep^ in HEK293T cells. Mutating C5aR1 residues from the murine to the human equivalent resulted in β-arrestin recruitment that is similar to human C5aR1 (Yadav et al. 2023). Consequently, caution is advised on the characterisation of agonists between species, as the same ligands may have different intended effects between mice and humans if both are given the same dose.

Signalling bias can also be modulated by changing the properties of agonists at specific positions. For example, a G_i/o_ protein agonist of C5a, BM213, has increased β-arrestin 2 bias following hydrophobic mutations of the peptide at P6 (Fig. 8B). This supports the involvement of hydrophobic interactions between the ligand and C5aR1 for modulation of β-arrestin recruitment. However, hydrophilic mutations have not been tested. Additionally, while most of the hydrophobic mutations result in increased β-arrestin 2 bias, the phenylalanine mutation did not have an impact.

In addition to the ligands which stimulate C5aR1 signalling, antagonists provide another layer of modulation by preferential inhibition of either G protein or β-arrestin recruitment. For example, PMX53, an orthosteric inhibitor for C5aR1, has greater inhibition of G_i_ activity compared to β-arrestin (Feng et al. 2023; Gorman et al. 2021). At the molecular level, PMX53 competes with C5a for the binding pocket of C5aR1. PMX53 inhibits C5aR1 activity by forming hydrophobic contacts with α-helices, H2 and H3, in C5aR1. Notably, this interaction is devoid of contact with E199 in C5aR1, supporting the importance of this residue to enable G_i_ recruitment (Fig. 8C).

On the other hand, Avacopan is an allosteric inhibitor of C5aR1. Avacopan is used to treat an autoimmune disease anti-neutrophil cytoplasmic antibody (ANCA)-associated vascularitis (Jayne et al. 2021). In this disease, complement is activated by autoantibodies targeted against cytoplasmic proteins in neutrophils. This causes the generation of C5a that binds to C5aR1 in neutrophils, resulting in neutrophil-mediated pathogenesis. Here, Avacopan has demonstrated bias in favour of β-arrestin recruitment, implying that β-arrestin signalling is potentially involved in the pathogenesis of C5a-activated neutrophils. Avacopan interacts with an allosteric site of C5aR1, capturing the conformation of H3, H4 and H5 predominantly through hydrophobic interactions (Fig. 8C). This indicates that the global conformation of C5aR1 plays an essential role in its phosphorylation and β-arrestin recruitment.

## MAC-Directed Disruption of Target Cells

So far, this review has only covered the molecular and structural insights for one-half of the terminal complement pathway consisting of C5a and C5a receptor signalling. Here, the second half of the terminal complement pathway will be explored, including the C5b effector pathway.

**Fig. 9:**
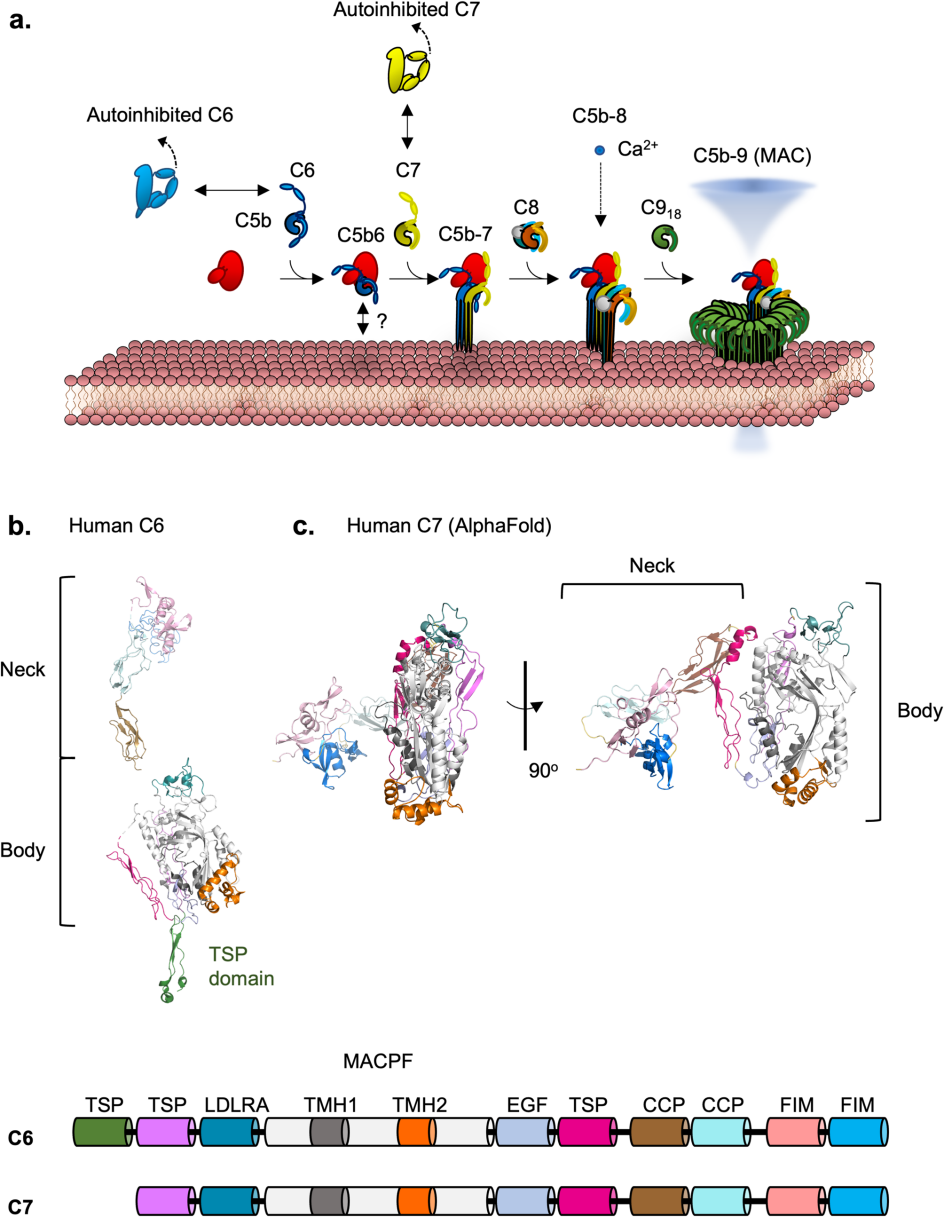
Overview of MAC formation and intrinsic regulatory steps. **a** Overall schematic of MAC assembly and the indicated intrinsic regulatory steps. This includes the autoinhibited and extended conformations of C6 and C7. Whether C5b6 interacts with the membrane is currently unclear and is indicated by the question mark. **b** Crystal structure of human C6 (PDB 3T5O), revealing the extended conformation. The tail, consisting of the TSP domain, is shown in dark green. **c** Predicted structure of human C7, revealing the autoinhibited conformation. The neck and body regions are indicated. Below is the domain map of C6 and C7

Cleavage of C5 produces two effectors, C5a and C5b. The latter component, C5b, is a precursor for the assembly of the cytolytic ring known as the membrane attack complex (MAC) or terminal complement complex (TCC). This C5b component is labile and requires immediate interaction with C6 to form a more stable C5b6. This is followed by stepwise sequential binding of C7, C8, and up to 18 copies of C9 that anchor, insert, and span the membrane, respectively (Fig. 9A). The final form is a hetero-oligomeric pore that assembles to puncture cell membranes. The complete MAC spans the membrane to form a pore lumen ~ 10 nm in diameter, killing microbes by either osmotic and ion flux or delivering secondary effectors such as lysozyme into the cell.

Pore formation and membrane insertion are achieved by the canonical MACPF/CDC domain, a domain that has been extensively characterised in proteins found in a range of different organisms (Rosado et al. 2008). This domain is found in C6, C7, C8 (α and β), and C9. Generally, the MACPF/CDC domain contains a bent L-shaped structure and is recognised to be responsible for both oligomerisation of pore subunits and membrane perforation. Each MACPF/CDC domain consists of two transmembrane hairpin (TMH) regions that unravel to form two long β-hairpins, which, upon oligomerisation insert into the membrane to form a β-barrel. Structures of the individual components, intermediates, and final pore are outlined in Table 3.

**Table 3:** List of MAC-related structures

	Structure	Accession Code	Resolution	Method	References
Monomeric components	Human C5	PDB: 3CU7	3.10 Å	X-Ray Crystallography	Fredslund et al. (2008)
	Human C6	PDB:3T5O	2.90 Å	X-Ray Crystallography	Aleshin et al. (2012a, b)
	Human C8αβγ	PDB: 3OJY	2.50 Å	X-Ray Crystallography	Lovelace et al. (2011)
	Murine C9	PDB: 6CXO	2.20 Å	X-Ray Crystallography	Spicer et al. (2018)
Intermediates	C5b6	PDB: 4A5W, 4E0S	3.50, 4.21 Å	X-Ray Crystallography	Aleshin et al. (2012a, b) and Hadders et al. (2012)
	sC5b-9_2_, sC5b-9_3_ Soluble MAC (sMAC)	PDB: 7NYC/7NYD EMD: 12646/12647/12648/12649/12650/12651	3.27/3.54 Å	Cryo-EM	Menny et al. (2021)
	C5b-8/C5b-9_2_/C5b-9_3_ with CD59 (CD59-MAC)	PDB: 8B0F/9B0H/8B0G EMD: 15779/15781/15780	3.00 Å/3.30 Å/3.30 Å	Cryo-EM	Couves et al. (2023)
Full pore	C9_22_ (PolyC9)	PDB: 6DLW EMD: 7773	3.90 Å	Cryo-EM	Spicer et al. (2018)
	C9_22_ (polyC9) with aE11	PDB: 8DE6 EMD: 28863	3.20 Å	Cryo-EM	Bayly-Jones et al. (2023)
	C5b-9_18_ (full MAC)	PDB: 6H03/6H04 EMD: 0106/0107/0109/0110/0111/0112/0113	5.60 Å	Cryo-EM	Menny et al. (2018)

### How Does the MAC Interact with the Membrane?

Single phospholipid bilayer model experiments have demonstrated membrane perturbations caused by the MAC, which are observed as early as the C5b6 step (Fig. 9A) (Liu et al. 1999; Marshall et al. 1996). It has, thus, been proposed that C5b6 interacts with modifications on the membrane surface in a lipid-specific manner, which includes ionic interactions, for example, with anionic sialic acid (Marshall et al. 1996). Presently, however, it is unclear which domains of C5b6 specifically coordinate with these lipid headgroups. Given that there is an extended protruding N-terminal domain in C6, known as a thrombospondin type-1 (TSP) domain, it has been hypothesised that this domain is involved in sensing lipids; however, there is currently no evidence to substantiate this hypothesis (Fig. 9B) (Liu et al. 1999; Parsons et al. 2019).

After C5b6 formation, the addition of C7 forms the C5b-7 intermediate that is stable on the membrane for up to 6–7 h via membrane anchoring by the C7 unit (Thai and Ogata 2005). Like C5b6, properties of the phospholipid heads also influence C7’s ability to anchor membranes (Liu et al. 1999). At this stage, it is hypothesised that the TMH regions of both C6 and C7 have unfurled and are partially inserted into the membrane as visualised by cryo-ET (Sharp et al. 2016). Sequence data demonstrates that the TMH regions of both C6 and C7 are shorter than C8 and C9 and are not physically long enough to completely span the membrane. Upon C5b-8 formation, C8αβ breaches the bilayer and forms an initial lesion large enough to enable ion leakage (Michaels et al. 1976; Zalman and Muller-Eberhard 1990). Finally, C5b-8 is the scaffold complex for multiple subunits of C9 to oligomerise, forming the large β-barrel pores.

## Regulation of MAC Formation

### Intrinsic Regulation of the MAC

Normally, pore-forming proteins contain a receptor binding domain to facilitate initial binding to its target. The MAC is unique to other pore-forming proteins as it has no known receptor-binding domain. This is advantageous as the MAC can target a wide array of pathogens. It is also known that this can lead to assembly on host cells, which is inhibited by various soluble and membrane inhibitors to trap MAC intermediates (Couves et al. 2023; Menny et al. 2021). However, as an unfortunate consequence, overwhelming levels of MAC or insufficient inhibition can lead to aberrant assembly on host membranes.

It is known through several crystallographic studies and cryo-EM structures that the initiation of the MAC is driven by the C-terminal domains in the neck region of C6 and C7 (complement control protein, factor I module domains, or CCP/FIMs) that interact with C5 (through the C345C domain) (Thai and Ogata 2004). The crystal structure of C6 reveals an extended seahorse-shaped conformation, where all the domains are in line with each other (Fig. 9B) (Aleshin et al. 2012a, b). In contrast to this extended conformation, recent cross-linking mass spectrometry demonstrates that alternative conformations of C6 exist in solution. In the cross-linking study, C6 adopts a compact conformation, where the neck region wraps around the MACPF/CDC core (Hevler et al. 2021). Surprisingly, the predicted structure of C7 follows a similar compact conformation (Fig. 9C) (Jumper et al. 2021). In this compact conformation, the domains on the neck of both C6 and C7 (CCP/FIM) are occluded from interacting with C5b, an interaction that is important in the initiation of MAC assembly. For C7, the neck may also protect from the oligomerisation interface of the MACPF/CDC domain from interacting with C6. Therefore, this conformation presents a potential intrinsic autoinhibitory mechanism.

C9, based on the structure of murine C9 and the modelling of human C9, has an unusually long first TMH1 region that threads through the protein to the extending oligomer interface. This location of the TMH1 region is a feature unique to C9 compared to other MACPF/CDC family members. Based on the TMH1 location, it was hypothesised that the TMH1 obstructs the oligomerisation interface of C9, which is only released upon the unfurling of TMH1, thereby acting as a “brake” in oligomerisation. Indeed, intramolecular disulfide trap mutants that restrained the TMH1 region of C9 prevented MAC oligomerisation. As such, it has been suggested that the rearrangement of this hairpin is required for oligomerisation that leads to a sequential insertion-oligomerisation mechanism (Spicer et al. 2018).

### CD59 Exhibits Two Different Inhibitory Mechanisms to Block C5b-8 and C5b-9 Perforation

Generally, host cells are protected against the MAC through the membrane-bound inhibitor CD59, the last line of defence against complement. CD59 is an approximately 20 kDa glycosylphosphatidylinositol (GPI) -anchored protein that traps membrane-bound MAC intermediates C5b-8 to possibly prevent initial insertion and prevent C9 from fully oligomerising. Therefore, cells are protected from MAC-mediated lysis by CD59.

**Fig. 10:**
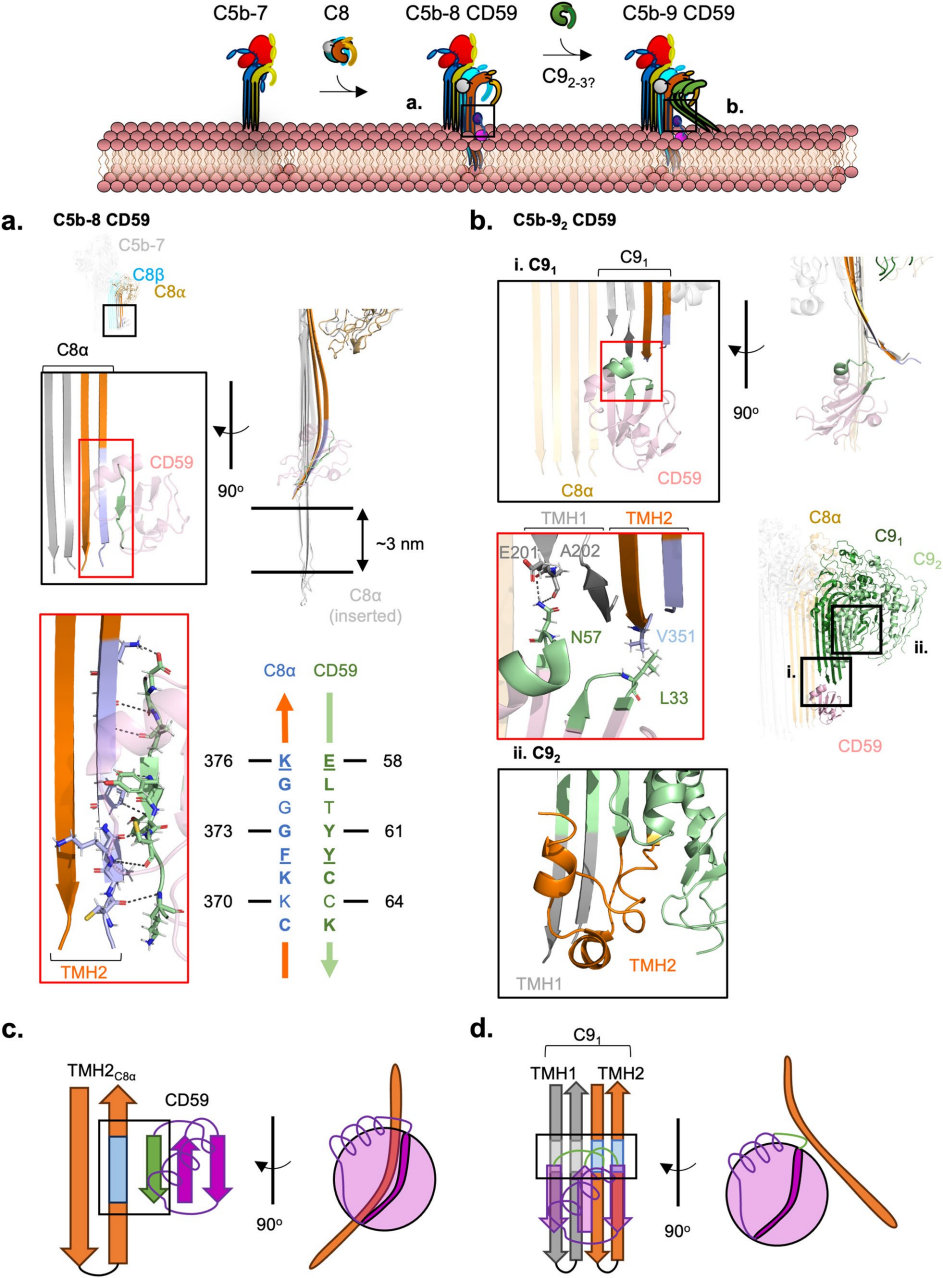
How CD59 inhibits MAC intermediates C5b-8 and C5b-9_2_/C5b-9_3_. Above is the illustrative overview of the steps behind how CD59 inhibits MAC assembly. **a** Detailed model behind CD59 inhibition of C5b-8 (PDB: 8B0F). One of the β-strands of CD59, in green, makes extensive contact with the TMH2 of C8α. The interacting residues are as shown. Residues contributing to backbone electrostatic interactions are in bold, whilst R-group interactions are underlined. The cross-section view illustrates the bending of inhibited C8α compared to an inserted C8α in light grey (PDB: 6H04). The approximate length of the phospholipid bilayer is indicated. **b** How CD59 inhibits the early C5b-9 intermediate (C5b-9_2_) (PDB: 8B0H). CD59 acts as a roadblock on full TMH1 & TMH2 unfurling of C9. (i) The interactions between the TMHs of C9 (in grey and blue) and CD59 (in green). (ii) The second C9 subunit (C9_2_), reveals a fully unravelled TMH1 in grey, but TMH2 remains in the helical form. **c** Simplified diagram of how CD59 interacts with C8α TMH2. **d** Simplified diagram of how CD59 interacts with the initial C9 subunit (C9_1_)

The most recent structural model of C5b-8 inhibited by CD59 reveals an interaction with the second TMH of C8α in C5b-8 (Couves et al. 2023). Here, the C8α TMH2 bends away from the phospholipid bilayer, potentially preventing membrane penetration. In this interaction, significant contacts occur between the peptide backbones of the C8α TMH2 and the central β-sheet of CD59 (Fig. 10A, C). However, this study still lacks a consensus on whether CD59 inhibits ion leakage. Molecular dynamic simulations reveal that, in CD59-inhibited C5b-8, C8β was partially inserted into the membrane but was impermeable to water (Couves et al. 2023). However, the extent to which C8α penetrates the bilayer is unclear. Other studies have supported that B-cells expressing CD59 block ion leakage by measuring the current potential (Farkas et al. 2002). However, these cells also possess active machinery such as endocytosis to prevent MAC leakage. Whether the same outcome applies to passive vesicles such as liposomes has not yet been tested experimentally.

The MAC/CD59 structure challenges the previous hypothesis regarding conserved CD59 inhibitory sites in C9 and C8α (Huang et al. 2006). Although C9 can bind to the oligomerisation interface of C8α, there is no clear sign of CD59 directly contacting the C9 hairpin to prevent membrane insertion. Instead, CD59 sits directly below and interferes with C9 membrane insertion by deflecting the hairpin away from the membrane, preventing membrane perforation (Fig. 10B, D).

The most recent investigation in the single-molecule kinetics of MAC formation looks at the rate of C9 oligomerisation after C5b-8 formation (Parsons et al. 2019). It was discovered that relative to C9 oligomerisation, the slowest step is the addition of the first C9, which potentially allows enough time for CD59 to effectively inhibit the full MAC. However, as the C5b-9/CD59 complex structure suggests, there is no complete inhibition of C9 oligomerisation despite CD59 binding to C5b-8.

### Conserved Inhibitory Mechanism in Soluble MAC

Through an unknown mechanism, freshly formed MAC intermediates (C5b-7, C5b-8 and C5b-9_1–4_) can fall off their intended target and attach to a nearby surface, a concept known as bystander lysis. Additionally, MAC intermediates that form on host cells can also be removed by active transport such as exocytosis. In either case, these soluble MAC intermediates (C5b-7, C5b-8 and C5b-9_2_/C5b-9_3_) are sequestered by soluble chaperones such as vitronectin and clusterin, forming another inhibited endpoint of MAC, the soluble membrane attack complex (sMAC) (Milis et al. 1993). These chaperones may function to prevent bystander lysis (Schmidt et al. 2016).

**Fig. 11:**
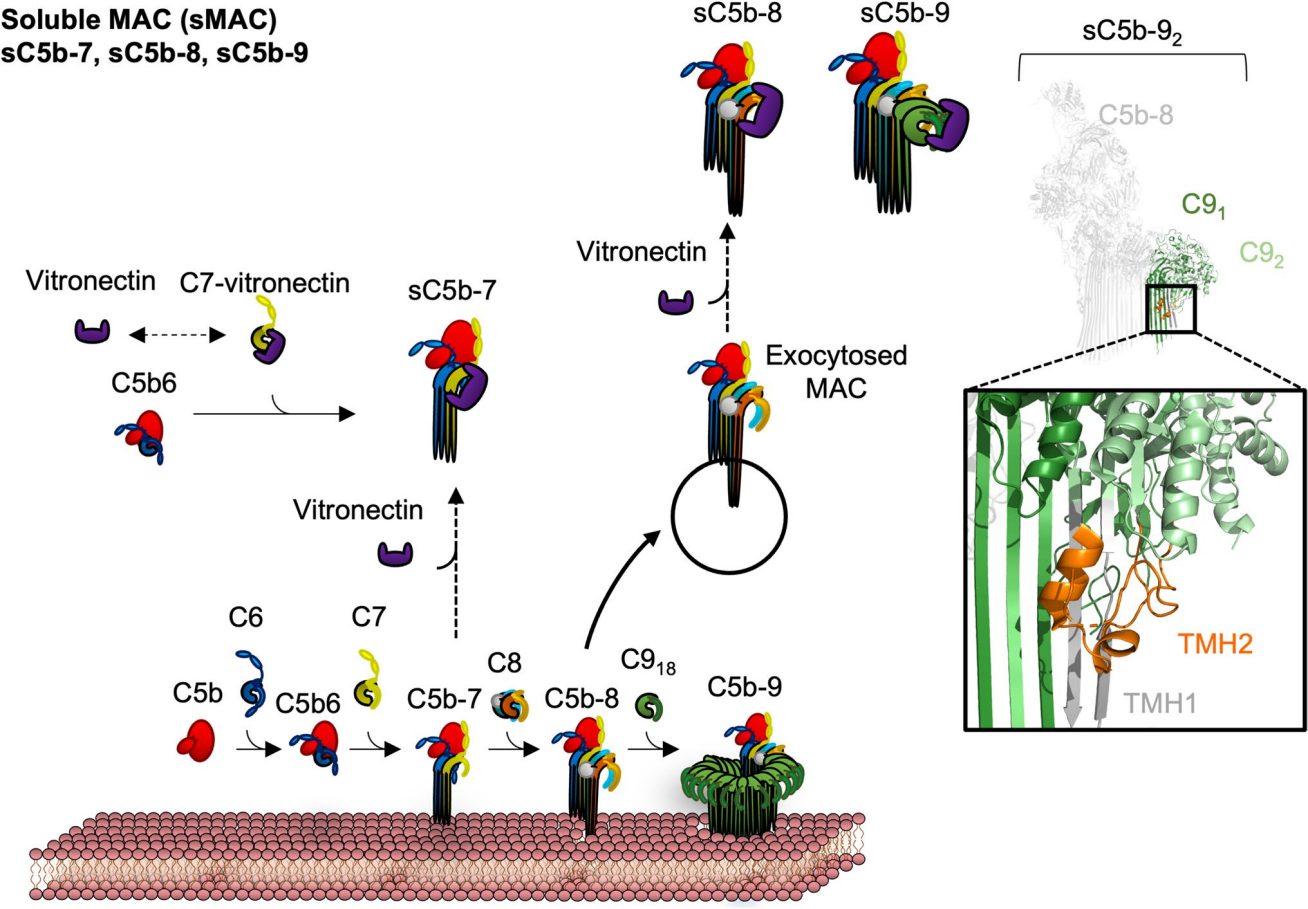
Potential mechanism behind the formation of soluble MAC and inhibition of MAC. Soluble inhibitors such as vitronectin reversibly bind to C7 but potentially have a much higher affinity for the non-anchored C5b-7 intermediate, forming soluble C5b-7 (sC5b-7). Later MAC intermediates, such as C5b-8 and early C5b-9 (containing ~ 2–3 C9 subunits), may be expelled by active transport such as exocytosis. These intermediates are captured by soluble inhibitors, forming sC5b-8 and sC5b-9. On the furthermost right is the structure of sC5b-9 (PDB: 7NYC), revealing a trapped intermediate conformation of the second C9 subunit (C9_2_). C9_2_ TMH1 (grey) is fully extended in the β-hairpin state, whilst C9_2_ TMH2 is α-helical

In the context of the C5b-9 version of sMAC, the cryo-EM map reveals that the chaperones capture the periphery of the components, including the ancillary domains and part of the MACPF/CDC domain (Menny et al. 2021). Like CD59-inhibited MAC, the structure of sMAC reveals a common regulatory mechanism of preventing the collapse of TMH2 after TMH1 unravelling (Fig. 11). Despite this, the steps leading to inhibition remain unknown. A recent study showed that clusterin forms a complex on monomeric C7 in healthy normal human serum (Massri et al. 2024). This provides a potential model where clusterin may reversibly bind to monomeric C7 but has a high affinity to C5b-7 upon formation of the C5b-7 intermediate (Fig. 11).

## Significance of the Terminal Complement Pathway in Inflammation

Both arms of the terminal complement pathway, that is, C5a/C5a receptors and the MAC, contribute to inflammation. The cellular effects of C5aR2 have been previously discussed (section "Elusive Role of C5aR2"). The cellular effects of C5aR1 stimulation by C5a have also been reviewed and vary in cell type and inflammatory disease (Schanzenbacher et al. 2023). Here, only a few examples are mentioned. In atherosclerosis and cholesterol crystal-mediated nephropathy, macrophages exhibit inflammatory phenotypes following cholesterol uptake. Following cholesterol crystal uptake of macrophages, autocrine C5aR1 activation in the mitochondrial membrane promoted reactive oxygen species generation and anaerobic metabolism to promote IL-1β expression (Niyonzima et al. 2021). Facilitating autoimmunity in transplants, C5aR1 activation in CD4 + T cells contributes to differentiation into follicular helper T cells and amplifies IL-21 expression (Verghese et al. 2018). Finally, in the mice model of Alzheimer’s disease, C5aR1 promotes astrocyte migration and microglial cell polarisation to release TNF-α and IL-1α (Carvalho et al. 2022; Hernandez et al. 2017). C5aR1 has been reported to restrict platelet-modulated vascularisation, making C5aR1 a potential therapeutic target for controlling angiogenesis (Nording et al. 2021).

MAC deposition on host cells has been correlated with inflammatory diseases. Indeed, inflammatory diseases have increased levels of the MAC, which is detected using diagnostic tools (Bayly-Jones et al. 2023). These inflammatory diseases are a result of excessive MAC formation where native safeguards, e.g., CD59, are overwhelmed, potentially resulting in ion leakage that can activate intracellular signalling pathways. Consequently, this leads to various effects on cells (Papadimitriou et al. 1994; Tegla et al. 2011). Evidence has emerged regarding MAC-mediated activation of signalling pathways and the cellular responses (Morgan 1992, 2016; Xie et al. 2020). For example, MAC deposition on epithelial cells induces mitochondrial depolarisation, leading to caspase and NLRP3 activation and ultimate apoptosis and inflammation (Nauta et al. 2002; Triantafilou et al. 2013). Mice studies reveal that the MAC mediates the release of cytokines, including IL-1β (Laudisi et al. 2013). Taken together, the combination of MAC and C5aR1/C5aR2 activation may result in a potential synergy of intracellular effectors, and examples of such will be discussed below.

### Emerging Evidence of Potential Crosstalk Between C5a-C5aR1/2 Axis and MAC Axis

**Fig. 12:**
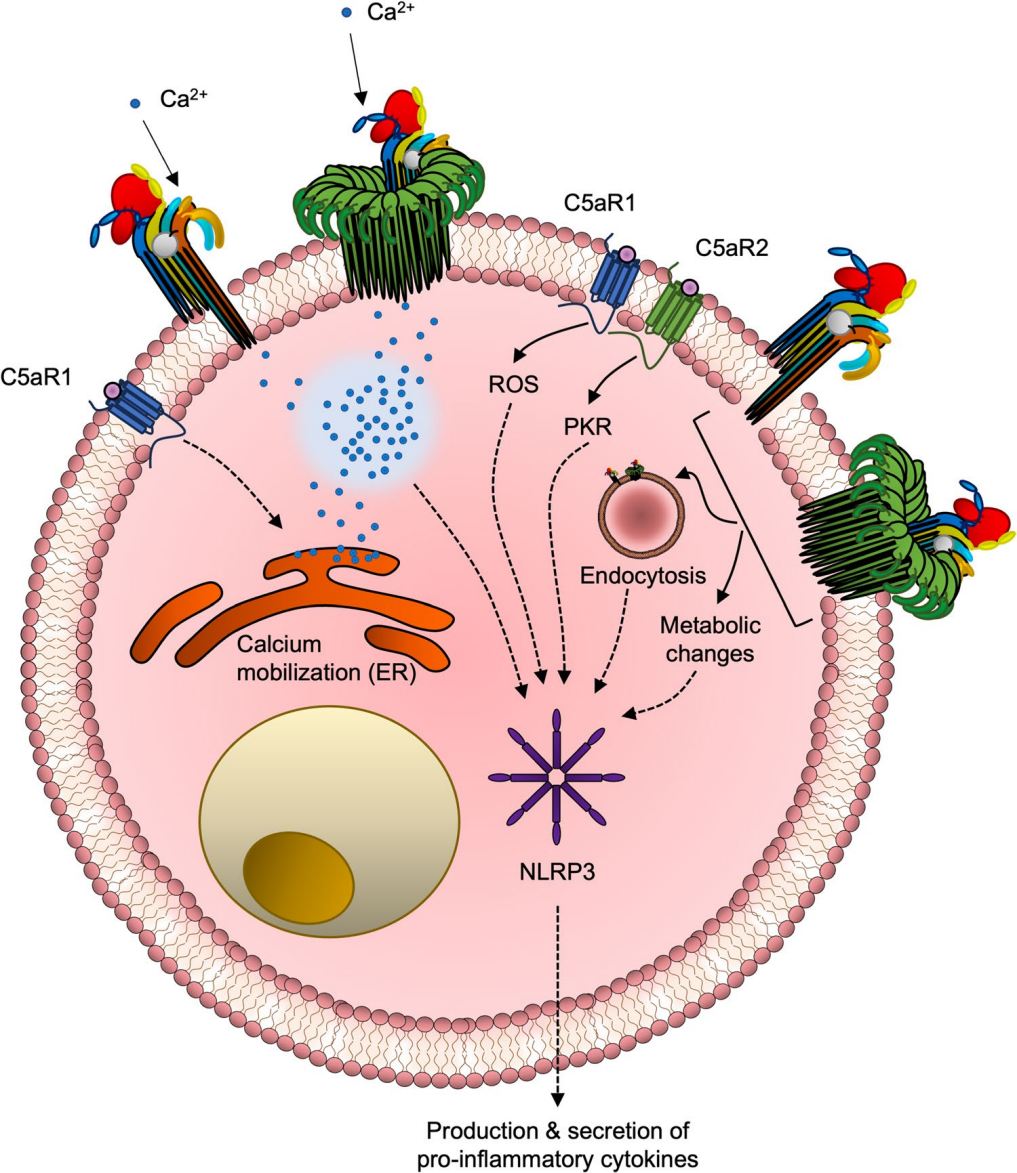
Contribution in C5aR1/C5aR2 activation and MAC formation on cell signalling. The left half of the cell illustrates potential mechanisms in Ca^2+^ mobilisation to the cytosol. C5aR1 activation induces Ca^2+^ mobilisation from the endoplasmic reticulum (ER), whilst the formation of MAC intermediates such as C5b-8 and C5b-9 enable Ca^2+^ leakage through the pore lumen. On the right-hand side of the cell is an illustration of the network of different signalling pathways leading to inflammasome activation. C5aR1 and C5aR2 stimulation by C5a results in pathways such as the generation of reactive oxygen species (ROS) and activation of protein kinase R (PKR). MAC deposition induces endocytosis and causes metabolic changes in the cell. Collectively, these pathways contribute to the activation of the NLRP3 inflammasome, ultimately resulting in the production and secretion of pro-inflammatory cytokines

Downstream signalling pathways following C5aR1/C5aR2 activation have been extensively reviewed (Lee et al. 2008; Schanzenbacher et al. 2023). When compared to MAC-mediated downstream signalling responses on host cells, some overlaps were noted. One of the potential outcomes of C5aR1 activation is the mobilisation of intracellular calcium from stores such as the endoplasmic reticulum. However, Gα_i_ is a regulator of adenylate cyclase, inhibiting ion channels and calcium influx and, therefore, limiting the pool of intracellular calcium for mobilisation. Given that the MAC also contributes to the influx of calcium, the combination of the two effectors potentially results in a synergised activity of calcium mobilisation (Fig. 12).

Both C5a and C5b pathways contribute synergistically to NLRP3 activation, resulting in the release of pro-inflammatory cytokines such as IL-1 (Fig. 12). It has recently been established that internalised MAC is required for NLRP3 inflammasome activation in HMDMs (Diaz-Del-Olmo et al. 2021; Jimenez-Duran et al. 2022; Xie et al. 2019). The evidence was supported by using purified MAC components to constitute the pore, excluding the possibility of C5a activating the inflammasome. It has been shown that C5aR1 contributes to NLRP3 activation in T cells, neutrophils and monocytes (Arbore et al. 2016; Samstad et al. 2014), however, the role of C5aR2 in NLRP3 activation is contentious. In T cells, C5aR2 downregulates NLRP3-mediated activity (Arbore et al. 2016), whereas in murine macrophages, C5aR2 promotes NLRP3 activation (Yu et al. 2019).

Whilst there is an overlap in the two systems activating NLRP3 activation, there is an apparent distinction in how these pathways activate NLRP3. C5aR1 activates NLRP3 through the generation of reactive oxygen species (ROS) (Arbore et al. 2016; Samstad et al. 2014). In macrophages, C5aR2 stimulation increases the transcription and expression of protein kinase R (PKR) (Yu et al. 2019). MAC activation, however, promotes NLRP3 activation either by potential interaction with the inflammasome following endocytosis or by indirect means such as promoting cellular metabolism resulting in the induction of mitochondrial stress or non-canonical NF-kB activation (Jimenez-Duran et al. 2022).

### Contribution of the Terminal Complement Pathway in Inflammatory Diseases

Terminal complement pathway-mediated inflammatory diseases are often convoluted and involve a multitude of immunometabolic pathways. The following Table 4 lists examples of inflammatory diseases involving both C5a receptor signalling and MAC formation. When exploring the underlying molecular biology behind the pathogenesis of certain inflammatory diseases, the two terminal complement pathways are likely to crosstalk and potentially work in synergy. Identifying the links between the two terminal complement pathways may be beneficial when elucidating the responsible terminal complement pathways for pathogenesis and becomes helpful when fine-tuning therapeutics. Here, three examples of terminal complement-mediated inflammatory diseases will be discussed to highlight the significance of the immune response at varying levels, from molecules to organs.

**Table 4:** List of inflammatory diseases involving both C5aR1/C5aR2 signalling and MAC formation

Disease	Potential terminal complement pathway contribution	References
Paroxysmal nocturnal haemoglobinuria	C5aR1 activation: contribution to thrombosis Membrane attack complex: intravascular haemolysis	Brodsky (2014), Hill et al. (2013) and Peacock-Young et al. (2018)
Atypical haemolytic uraemic syndrome	C5aR1 activation: platelet aggregation, promotion of thrombosis Membrane attack complex: thrombotic microangiopathy development in the kidney	Aiello et al. (2022) and Smith-Jackson et al. (2025)
Antiphospholipid syndrome	C5aR1 activation: contribution to thrombosis in mice, mediating oxidative burst in neutrophils Membrane attack complex: contribution to platelet-leukocyte aggregates and thrombotic occlusions	Fischetti et al. (2005), Girardi et al. (2003), Redecha et al. (2007) and Romay-Penabad et al. (2007)
Systemic lupus erythematosus	C5aR1 activation: mitochondrial fission in podocytes, pro-inflammatory cytokine release (IL-6, TNF-α) (rat model of Thy-1 nephritis), recruitment of neutrophils and macrophages to kidneys (mice) Membrane attack complex: localisation on glomeruli and peritubular basement (kidneys), basement membrane zone of cutaneous lesions (skin)	Bao et al. (2005), Biesecker et al. (1981), Helm and Peters (1993), Ji et al. (2016) and Ye et al. (2024)
Aged macular degeneration	C5aR1 activation: recruitment of mononuclear phagocytes to choroid-retinal pigment epithelium interface, promotion of choroidal neovascularisation, migration of retinal pigment epithelial cells Membrane attack complex: localised deposition on the choriocapillaris, Bruch’s membrane	Chirco et al. (2016), Mullins et al. (2014) and Nozaki et al. (2006)
Rheumatoid arthritis	C5aR1 activation: leukocyte migration to synovial fluid Membrane attack complex: increased levels of sMAC in the synovial fluid	Hornum et al. (2017) and Morgan et al. (1988)
Atherosclerosis	C5aR1/C5aR2 activation: Recruitment of macrophages to atherosclerotic lesions, production of pro-inflammatory cytokines in mice C5aR2 activation: production of pro-inflammatory cytokines (IL-1β, TNF-α), enhancement of integrin expression Membrane attack complex: increased deposition on the plaque, smooth muscle cell cytolysis in advanced plaques	An et al. (2016, 2014), Lewis et al. (2010), Selle et al. (2015) and Vijayan et al. (2014)
Amyotrophic lateral sclerosis (ALS)	C5aR1 activation: macrophage recruitment into skeletal muscles Membrane attack complex: increased levels of sMAC in plasma	Bahia El Idrissi et al. (2016), Mantovani et al. (2014) and Wang et al. (2017b)
Alzheimer’s disease	C5aR1 activation: microglial cell mediated inflammation Membrane attack complex: destruction of synapses in mice	Carpanini et al. (2022) and Schartz et al. 2024)

#### Paroxysmal Nocturnal Haemoglobinuria (PNH)

**Fig. 13:**
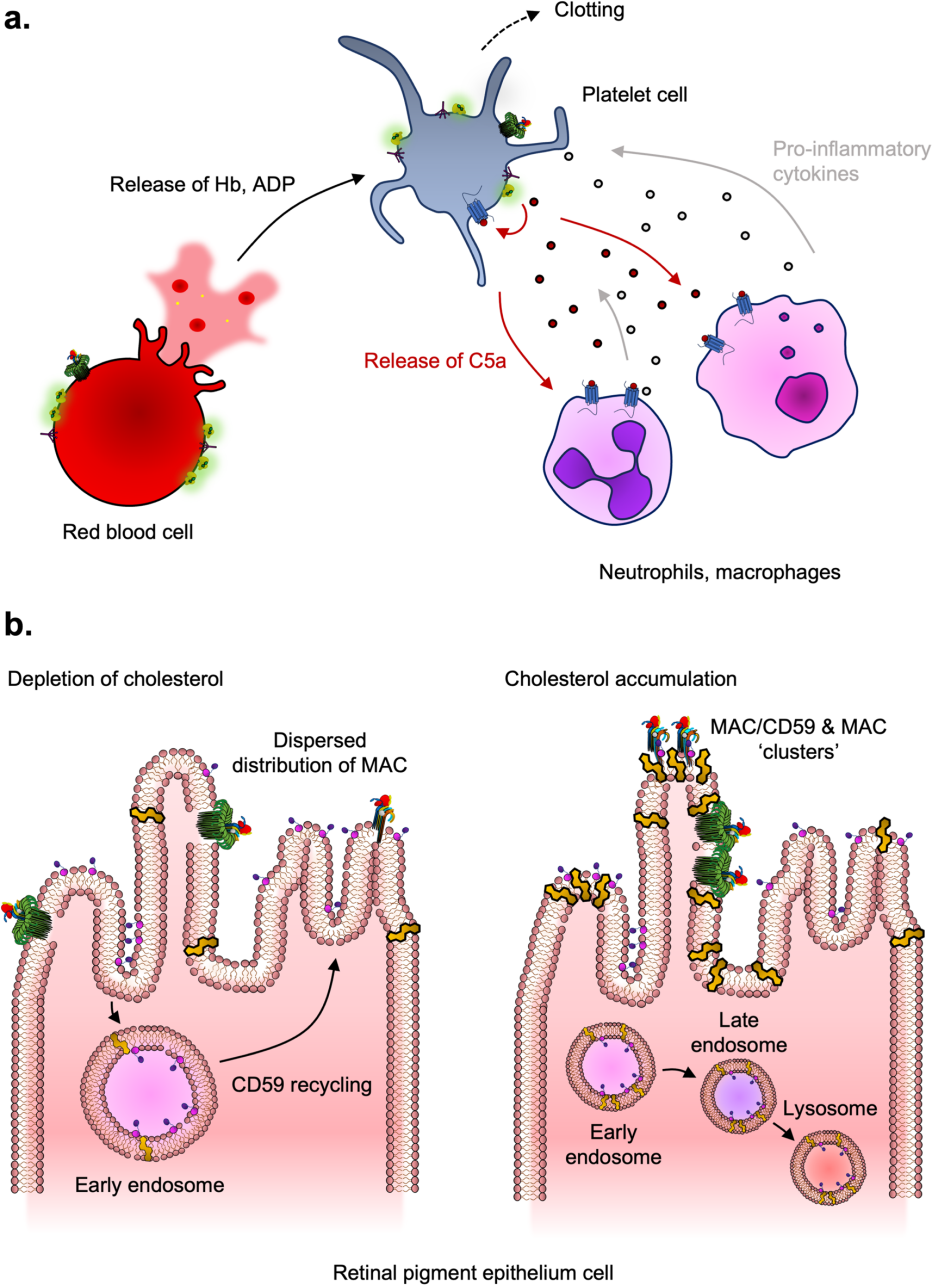
Interplay of MAC deposition and C5a/C5aR1 signalling in PNH, and mechanism behind distribution of membrane CD59 on retinal pigment epithelium cells. **a** In PNH, MAC induces osmotic influx in red blood cells, resulting in its bursting and subsequent release of haemoglobin and metabolites such as ADP. Platelet cells are subject to ADP from red blood cells, MAC formation, and C5aR1 stimulation. This contributes to the clotting symptoms of PNH, as well as pro-inflammatory responses resulting from the stimulation of leukocytes such as neutrophils and macrophages. The release of cytokines by these leukocytes stimulates platelets, forming a positive feedback loop of inflammation and clotting. **b** The role of cholesterol on the distribution of CD59 in retinal pigment epithelium cells involved in aged macular degeneration (AMD). On the left, cholesterol depletion promotes CD59 recycling. Furthermore, MAC formation is dispersedly distributed. By comparison, a cholesterol-rich membrane environment may contribute to the formation of the CD59 lipid rafts. In addition, the MAC tends to form in clusters

PNH comes about from a somatic mutation of the PIG-1 gene in myeloid cell lines. These cell lines lack the PIG-1 enzyme required for the GPI-anchoring of proteins, including CD59. Therefore, cells of myeloid origin are deficient in CD59 and are vulnerable to MAC-mediated lysis (Boccuni et al. 2000). Haemolysis results in the release of haemoglobin and various metabolites such as ADP. Neighbouring platelets, on top of lysis, can also react to released erythrocyte constituents and activate the clotting pathway, resulting in thrombosis. Indeed, treatment with eculizumab reduced the incidence of thrombosis, indicating that the activation of C5 is involved in the thrombotic pathway (Hillmen et al. 2007). The importance of MAC formation in platelet activation is reflected in a study where MAC-mediated haemolysis is required for the release of ADP to prime and activate platelet cells (Fig. 13A) (Mannes et al. 2023).

While the symptoms mentioned depend on MAC-mediated lysis, other considerations, such as the concomitant release of C5a, have not been extensively studied. C5a may play a contributing and facilitative role in thrombosis following the activation of platelets mediated by MAC. Activated platelets may present a hyperactive phenotype following C5a-C5aR1 activation, a phenomenon that has been observed for other diseases such as COVID-19 (Apostolidis et al. 2022; Caillon et al. 2022). C5a can also potentially bind to neighbouring neutrophils and macrophages in the vasculature, resulting in its activation and release of pro-inflammatory cytokines such as IL-6. IL-6 is known to contribute to prolonged thrombosis by upregulating thrombin synthesis (Dowling et al. 2022; Zhang et al. 2020b). The significance of thrombosis applies to a small population where their symptoms are not manifested by significant haemolysis (Griffin et al. 2019).

#### Atypical Haemolytic Uraemic Syndrome (aHUS)

Another rare disease, aHUS, also known as complement-mediated haemolytic uraemic syndrome, has similar symptoms to PNH, including thrombosis and haemolysis, although it is also characterised by renal dysfunction. The primary cause behind aHUS regarding complement involves overwhelming alternative pathway complement activation, leading to the aberrant formation of C5 convertases. Elements responsible for this include genetic mutations of alternative pathway-regulating factors, rendering them inactive, and mutations on the C5 convertase component Factor B that result in an overactive convertase (Goicoechea de Jorge et al. 2007).

Differential effects between MAC mediated and C5a-C5aR1 mediated effectors have been studied in a Factor H mutant mice model of thrombotic microangiopathy (TMA), a hallmark of aHUS (Ueda et al. 2019). The MAC has a larger contributing role to the pathogenesis than C5aR1, as mice with defective Factor H (a regulator of complement activation) and deficiency of C6 have a larger survival rate compared to mice with defective Factor H and deficiency of C5aR1. In addition, knockouts of the terminal components on defective Factor H mice have different effects depending on the location of thrombosis. For macro vessels in the kidney, liver, lung and spleen, only C5aR1 knockout mice ameliorates thrombosis, whilst for TMA, only C6 and C9 knockout mice ameliorate thrombosis. Here, only glomerulus tissue was analysed for the latter observation. Whether other capillary-rich tissues (e.g., alveoli of the lung) are also affected is unclear.

#### Aged Macular Degeneration (AMD)

Aged macular degeneration (AMD), a disorder that commonly occurs in the older population, consists of excessive complement activation in the retina, causing a loss of vision. This is known to be associated with polymorphisms of FH that lead to reduced or loss of alternative pathway inhibition (Tzoumas et al. 2021). To investigate the involvement of terminal complement, studies have explored treating human retinal pigment epithelium (RPE) cells with normal human serum, thereby initiating complement and MAC on the cells. RPEs were treated with sub-lytic MAC (by definition, causing < 5% cytotoxicity) to study coping mechanisms of counteracting the MAC (Lueck et al. 2011).

However, as normal human serum was used, there is potential for C5a-C5aR1 signalling on the RPEs. Indeed, murine RPEs express C5aR1 and react to C5a, resulting in increased migration (Llorian-Salvador et al. 2022). To determine the weighting of C5a-C5aR1 contribution in AMD, an anti-C5a antibody was used to block this pathway in a heterozygous FH-knockout experimental mice model of AMD (Toomey et al. 2018). While anti-C5a therapy decreases infiltration of neutrophils and monocytosis, there is retention of inflammatory features, including an increase in thickness on sub-RPE basal laminar deposit, RNA expression of inflammatory markers compared to the control (Toomey et al. 2018). The retention of inflammation suggests that other factors are involved, potentially including MAC formation.

RPEs express CD59 to protect themselves against the MAC. On top of the known molecular mechanism of CD59 inhibition, it is also interesting to explore whether the distribution of CD59 in host cells influences its activity against the MAC. Together with previous studies, membrane-bound CD59 can also undergo endosomal pathways for either recycling or lysosomal degradation. The manipulation of surface cholesterol also influences the fate of GPI-anchored CD59. In the case of RPEs, manipulation of cholesterol levels has impacted levels of surface CD59 (Tan et al. 2016) (Fig. 13B). Consequently, a decrease in surface CD59 provides less protection at the membrane surface against the MAC.

Furthermore, the presence of cholesterol also influences the distribution of CD59 on the membrane. Cholesterol contributes to the formation of lipid rafts, which are functional domains on the membrane surface that play a role in cell signalling and membrane protein dynamics (Kusumi et al. 2020). In part, lipid rafts are formed from cooperative interactions between cholesterol and both unsaturated and saturated acyl chains of the lipids. CD59 has been found as a higher-order oligomer in enriched cholesterol regions (Koyama-Honda et al. 2020). Furthermore, CD59 dimers formed from the lipid rafts help in recruiting downstream signalling proteins (Suzuki et al. 2012).

Reflecting this, cholesterol also influences the deposition of MAC in Chinese Hamster ovary cells (Couves et al. 2023). In the presence of cholesterol, the MAC tends to form in clusters, whilst in cholesterol-depleted cells, the MAC is more sparsely distributed (Fig. 13B). There has been evidence that MAC clusters co-localise with cholesterol and that both CD59 rafts, as well as MAC deposition, are required for Ca^2+^ mobilisation (Suzuki et al. 2012). Based on the existing findings that CD59 may block MAC-mediated ion leakage, other factors might contribute to an increase in cytosolic Ca^2+^, such as the potential for MAC-mediated intracellular signalling. Overall, these findings provide fresh insights regarding alternative ways that MAC, CD59, and the contributing cholesterol may trigger pro-inflammatory responses in RPEs.

## Emerging Therapeutics to Selectively Target Terminal Complement Pathway Effectors

To treat terminal complement inflammatory diseases, therapies have been developed and have been extensively reviewed (West et al. 2024; Zelek et al. 2019). The most common monoclonal antibody that is used to treat PNH and aHUS is Eculizumab. Eculizumab prevents C5 cleavage by binding to the cleavage site of C5 (Jore et al. 2016; Schatz-Jakobsen et al. 2016). Given the tremendous success of Eculizumab, there have been efforts to improve its efficacy, leading to the generation of Ravulizumab (Lee et al. 2019). However, there are a few who are resistant to the inhibitor. This may arise from genetic variations of C5, different conformations of C5, or residual cleavage of C5 (Harder et al. 2017; Nishimura et al. 2014).

Certain terminal complement diseases can be treated by selectively inhibiting one arm. Concerning PNH, haemolysis can be treated by selectively inhibiting MAC formation. Furthermore, MAC formation has greater importance in the pathogenesis of experimental autoimmune encephalomyelitis, given that C5aR1 blockage does not protect rats from the disease (Michailidou et al. 2018; Morgan et al. 2004). Recent discoveries of monoclonal antibodies inhibiting the MAC that target C6 and C7 have opened new avenues in this light, which have shown to be effective in other inflammatory diseases, including myasthenia gravis (Gytz Olesen et al. 2023; Lekova et al. 2022; Lin et al. 2020; Zelek and Morgan 2020). The most recent structure of the C6 inhibitory antibody reveals binding to the FIM domain of C6, highlighting the importance of this domain to initiate the MAC (Gytz Olesen et al. 2023). There have also been developing therapeutics that recognise neoepitopes on MAC intermediates such as C5b6. However, these antibodies have failed to prevent MAC formation on membranes (Stach et al. 2021). Together, these therapies not only help tackle diseases whose pathogenesis is predominantly driven by MAC but also retain the C5a-C5aR1/C5aR2 axis, which may be important for priming innate and adaptive immune cells for fighting infection (Desai et al. 2023; Kim et al. 2004). Diseases that are predominantly driven by C5aR1 can be treated with antagonists such as Avacopan for ANCA-associated vasculitis, helping to retain the MAC for infection.

## Concluding Remarks

This review has provided the current dynamic landscape behind how the terminal complement pathway interacts and modulates the membrane environment, ranging from the fine molecular detail to the systemic level. Recent insights into the structures of C5a and other ligands binding to C5aR1 have provided the molecular basis for receptor activation and biased agonism. Notably, the structure of C5a desArg binding to C5aR1 coupled with the G_i/o_ protein has yielded insights behind the dramatic loss of affinity of the ligand to the receptor following the removal of the arginine. However, when comparing the key C5a-interacting residues of C5aR1 to C5aR2, these residues are almost identical in C5aR2. This analysis raises questions on whether C5a similarly binds to C5aR2. Additionally, the structural differences that explain the higher affinity of C5a desArg to C5aR2 remain to be solved. Other than ligand binding, the different phosphorylation motifs encoded on the C-terminus between C5aR1 and C5aR2 may explain their differences in β-arrestin-mediated function. The exact intracellular signalling pathways activated by β-arrestin (1 or 2) following C5aR2 stimulation have yet to be further investigated. Recent structures of inhibited MAC reveal distinct mechanisms of inhibition by CD59 and vitronectin/clusterin. Both inhibitors destabilise the growing pore by trapping an intermediate form, ultimately preventing further oligomerisation.

Studying the role of the terminal complement pathway is a big challenge in inflammatory diseases. Firstly, both arms of the terminal complement pathway have overlapping contributions in initiating pro-inflammatory responses. An example is where C5a receptor activation and MAC formation can activate their unique intracellular signalling pathway that converges to NLRP3 activation. Distinguishing the roles of each pathway in the exemplified diseases shows that while it is possible that both arms concomitantly occur at the inflammatory site, one arm may dominate over the other. This may depend on the availability of surface C5a receptors, membrane distribution of CD59, or the presence of unidentified inhibitory mechanisms. Another problem is the pleiotropic effects exerted by cells stimulated with C5a to mediate inflammation. The recent structural insights of C5aR1 may help develop C5aR1-specific β-arrestin or G protein recruitment-biased agonists, which will clarify the connection of C5aR1-mediated signalling pathways activated with the cellular effect. While C5aR1 antagonists have already been used to elucidate the importance of C5aR1, the generation of a C5aR2-specific antagonist may help delineate functional differences between the two receptors.

Finally, delineating the two arms and their respective roles in complex inflammatory diseases may assist in selectively inhibiting one arm of the complement pathway rather than inhibiting C5 cleavage. Tools such as C6- or C7-knockout mice or mice treated with MAC-specific inhibitory antibodies may help investigate the importance of the MAC in disease models in vivo. Altogether, these efforts would enable the treatment of rare complement diseases while also providing innate immunity against infection.

## Data Availability

No datasets were generated or analysed during the current study.
